# Chemical composition and studying the possible neuroprotective effect of iridoids-rich fraction from *Pentas lanceolata* leaves using rotenone model of Parkinson’s disease in mice

**DOI:** 10.1007/s10787-024-01509-9

**Published:** 2024-07-04

**Authors:** Ahmed H. Afifi, Heba-Tollah M. Sweelam, Marwa E. El-Shamarka, Hisham A. Orban, Wessam H. Elesawy, Maki Nagata, Kuniyoshi Shimizu, Howaida I. Abd-Alla

**Affiliations:** 1 Pharmacognosy Department, Pharmaceutical and Drug Industries Research Institute, National Research Centre, 33 El Bohouth St. (Former El Tahrir St.), Dokki, 12622 Giza Egypt; 2 Chemistry of Natural Compounds Department, Pharmaceutical and Drug Industries Research Institute, National Research Centre, 33 El Bohouth St. (Former El Tahrir St.), Dokki, 12622 Giza Egypt; 3 Narcotics, Ergogenics and Poisons Department, Medical Research and Clinical Studies Institute, National Research Centre, 33 El Bohouth St. (Former El Tahrir St.), Dokki, 12622 Giza Egypt; 4 Biochemistry Department, Department of Medical Biochemistry, Medical Research and Clinical Studies Institute, National Research Centre, 33 El Bohouth St. (Former El Tahrir St.), Dokki, 12622 Giza Egypt; 5 Pharmacology and Toxicology Department, College of Pharmaceutical Sciences and Drug Manufacturing, Misr University for Science and Technology (MUST), 6 October, Egypt; 6 Department of Agro-Environmental Sciences, Graduate School of Bioresource and Bioenvironmental Sciences, Kyushu University, Fukuoka, 819-0395 Japan; 7 Kyushu University Institute for Asian and Oceanian Studies, Kyushu University, Fukuoka, 819-0395 Japan

**Keywords:** *Pentas lanceolata*, Plant iridoids, Parkinson's disease, Motor functions, Behavioral tests Rotenone

## Abstract

Parkinsonism is an age-related neurodegenerative illness that affects motor coordination leading to loss of dopaminergic neurons. Many medications are used for the treatment of Parkinson’s disease but are only symptomatic and have a limited effect on the progression of this ailment. Therefore, bioactive compounds which derived from plants have been examined for their ability to improve the neuronal damage and cell death happened in parkinsonian patients. In this study the iridoids-rich fraction isolated from *Pentas lanceolata* (PIRF) leaves was investigated for its phytoconstituents. Seven iridoids (**1**–**7**) and one flavonol diglycoside (**8**) were isolated, and their chemical structures were achieved by ^1^H and ^13^C nuclear magnetic resonance and ESI–MS spectral data. Compound** 1** (6β,7β-epoxy-8-*epi*-splendoside) and **5** (gaertneroside) were isolated for the first time from *Pentas* genus as well as compound **8** (kaempferol-3-*O*-robinobioside). The current study aims to investigate the possible anti-parkinsonian effect of PIRF using a rotenone model of Parkinsonism in mice. Behavioural tests (wirehanging, stair and wooden-walking tests) were done to examine the motor coordination in mice after treatment. Biochemical and histopathological examinations for brain striatum in different groups were also evaluated. Results revealed that rotenone-treated mice had poor motor functions described by depletion of dopamine and Ach levels, a significant increase in proinflammatory cytokines, IL-1B, TNF-α and Mcp-1 and oxidative biomarkers with subsequent reduction in antioxidant mediators. Disorganization of striatum, degenerated neurocytes, slight vacuolation, shrunken neurons with pyknotic nuclei and apoptotic cells are displayed by histopathological examinations. Treatment with PIRF ameliorates the neurodegeneration-induced by rotenone in the brain of mice. The anti-parkinsonian effect of PIRF could be attributed to their bioactive constituents of iridoids.

**Supplementary Information:**

The online version contains supplementary material available at 10.1007/s10787-024-01509-9.

## INTRODUCTION

One of the most sever disabling disorders in the old age is Parkinson's disease (PD). Globally, PD is the second neurodegenerative disease after Alzheimer's disease in people above 65 years old (McGregor and Nelson 2019; Alabi et al. 2019). The number of patients suffering from PD is increasing year by year and this cause a serious social problem and the development of targeted and effective agents for their treatment remains challenging (Jin et al. 2023). In PD, Lewy bodies (eosinophilic intracytoplasmic inclusions) are formed due to accumulation of α-synuclein synaptic protein, causing neuronal death. Moreover, dopaminergic neurons’ loss in the substantia nigra is considered the main hallmark of this disease. Several investigations suggest a strong association between β-amyloid deposition in brain and cognitive impairment explored in PD (de la Mora et al. 2019; El- Shamarka et al. 2023). Rotenone, a naturally occurring insecticide is conveyed as a potent neurotoxic agent. It is commonly used in the research field for induction of neuronal loss in animal models of PD (Abdel-Salam et al. 2014; Farid et al. 2024). Experimentally, rotenone administration is accompanied with cerebral neuroinflammation and oxidative damage (Alabi et al. 2019).

However, due to the existence of the blood–brain barrier (BBB), classical effective drugs, such as anti-inflammatory drugs (non-steroidal), have limited therapeutic effects on neurological diseases (Sweelam et al. 2017; Zhou et al. 2023). Therefore, the development of new drugs is of great significance for the prevention or treatment of nervous system diseases. In recent years, with the advance of technology and science, more and more researchers have turned their attention to natural products specially to plants rich in certain class of naturally occurring monoterpenoids; iridoids (Wang et al. 2020; Zhou et al. 2023; Abd-Alla et al. 2024). The use of plant extracts in treatment of Parkinson’s disease is rising worldwide to decrease the serious side effects of the chemical medications (Grover et al. 2023; Zhou et al. 2023). Plant fractions and/or extracts have antioxidant and anti-inflammatory properties that are very effective in treatment of PD (Grover et al. 2023).

*Pentas lanceolata* is known as Egyptian Star cluster, and belongs to the family Rubiaceae (Sweelam et al. 2017). In one of the flowering plants of family Rubiaceae, the monoterpene iridoids having a skeletal structure of methylcyclopentan-[*C*]-pyran. These compounds are widely distributed as glycosides (Abd-Alla et al. 2024). These plant metabolites are divided into C_10_, C_9_ and C_8_-iridoid glycosides as skeletal structural groups, based on the atoms of carbon number actually present in the main structure of their aglycone part. Most of plant iridoid glycosides contain glucose unit as sugar moiety and hence are called iridoid glucosides (Sweelam et al. 2017; Zhou et al. 2023). These compounds are isolated from plants by extraction followed by application of analytical chromatographic methods for their purification (Abd-Alla et al. 2017; Fahmy et al. 2020). Many of the iridoids (about 3000) reported with a varied biological activities such as immunomodulatory, antidiabetic, hepatoprotective, anti-inflammatory, anticancer/antitumor, strong antioxidant, neuroprotective, antiviral, and antimicrobial activities (Abd-Alla et al., 2020, Abd-Alla et al. 2024; Dinda et al. 2019).* Pentas lanceolata* (Forssk.) is used in the treatment of tropical diseases such as abdominal cramps, ascariasis and lymphadenitis (Sweelam et al. 2017; Saad et al. 2024a, b).

Iridoids and their glycosides constitute the major secondary metabolites in *P. lanceolata*, especially in the aerial parts (Venditti et al. 2015; Sweelam et al. 2017). These fractions previously displayed a wide array of pharmacological activities such as cardiovascular, hepato-protection, hypoglycemic, anti-mutagenic, antispasmodic, anti-tumor, antiviral, immunomodulation, and purgative effects (Wang et al. 2020; Sweelam et al. 2017).

The current study aims to investigate the potential anti-parkinsonian effect of the iridoids-rich fraction isolated from *Pentas lanceolata* (PIRF) leaves against experimental rotenone-induced PD in mice dependent on biochemical, behavioral, and histopathological evidence based on the available data. PIRF effects in PD models have not yet been examined.

The present study underlies the mechanisms of PIRF in rotenone -induced PD model and evaluates the protective effects of the investigated fraction. A remarkable protective role of PIRF in parkinsonian mice has been recorded. This provides increased dopamine availability and neuronal protection via assessing various parameters as well as restoration of behavioral function. These findings suggest that PIRF has potential benefits for treating PD and PD-like diseases as one of the main neurodegenerative diseases. Also, the investigation of the chemical constituents of the plant in the current study has illustrated for the first time and the isolation of the flavonol diglycoside; compound **8** (kaempferol-3-*O*-robinobioside); a rare class of compounds present in the genus of *Pentas*.

## MATERIALS AND METHODS

### General

Medium-pressure liquid chromatography (Pure C-850 Flash prep®, Buchi, Switzerland) with UV-ELSD detection connected to reversed phase flash columns (Flash pure C18, 40 μm, 4 and 12 g) was used for final purification. DRX-600 spectrometer (Bruker Daltonics, USA) was used to record nuclear magnetic resonance (NMR) spectra. “Waters” 3100 “USA", TQ Detector (Acquity ultra performance LC), Mass lynx V 4.1 was used for ESI–MS spectra.

### Drugs, chemicals and kits

Rotenone was obtained from Sigma-Aldrich and it was dissolved in DMSO (dimethyl sulfoxide). From Sigma-Aldrich in USA, other reagents and chemicals of analytical grade were purchased. There were five Eliza kits obtained from Sunlong Biotech Co. LTD in this study.

### Plant material and botanical identification

The leaves of *Pentas lanceolata* were collected from Al-Orman Botanical Garden in November 2021, Giza, Egypt. The plants were botanically identified by Treas Labib, Herbarium Section, El-Orman Botanical Garden, Giza, Egypt. The identification was confirmed by Dr Reem Sameer Hamdi, professor of plant taxonomy and flora, Faculty of Science, Botany Department (Cairo University). In our laboratory, a sample of the studied plant is being maintained under the accession number (No. 2021-P.5).

### Extraction and isolation of compounds

**Fig. 1:**
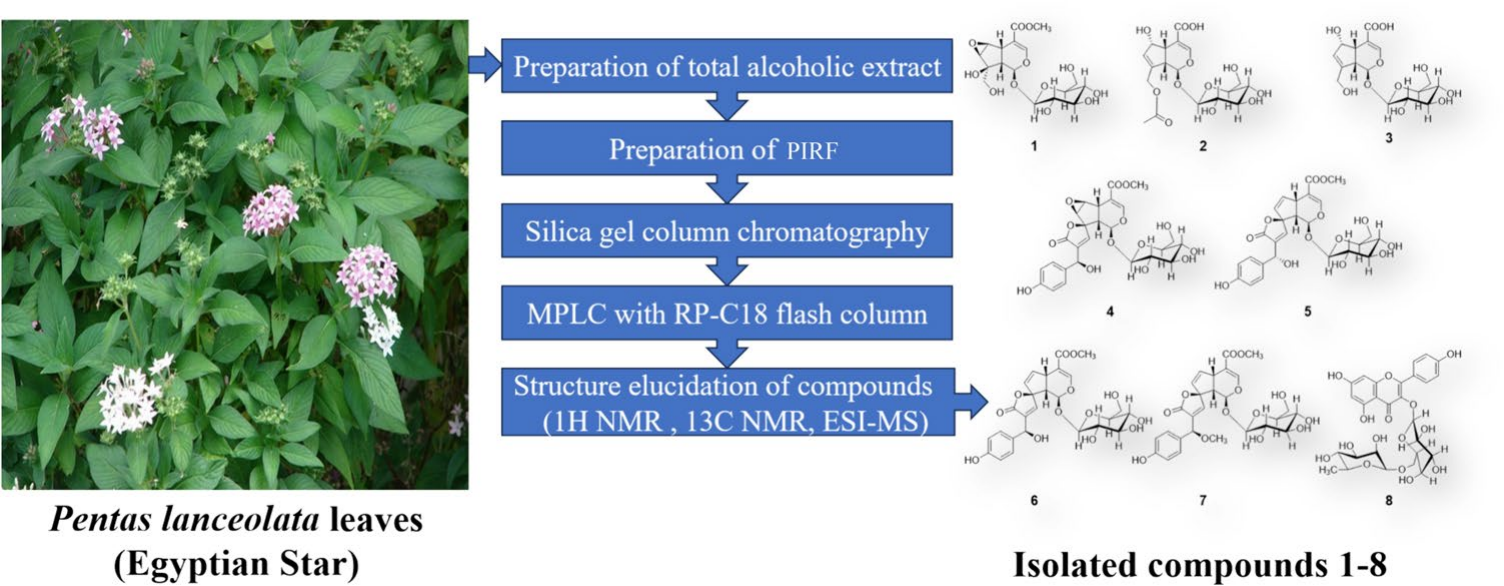
Design of extraction and isolation of compounds of iridoids-rich fraction from *Pentas lanceolata * leaves (PIRF)

The extraction was carried out according to the method of Abd-Alla et al. (2022) and the preparation of iridoids-rich fraction (PIRF) has been performed according to our previous work (Fahmy et al. 2020). A brownish-green dry fraction (PIRF, 17.32 g) was stored at 4 °C until use. Part of PIRF (11 g) was subjected to column chromatography (80 cm × 80 mm packed with 320 g silica gel 60–120 mesh Merck) eluted with an elution system of dichloromethane: methanol (9:1) to give rise of two main fractions (A and B). Fraction A (400 mg) rechromatographed on silica gel (22.0 g). Methanol/ chloroform system was used as mobile phase. The column was conditioned with methanol/chloroform 0.5:9.5, v/v and the chromatographic run started with mixture 9: 1 (v/v). Gradually, the polarity was increased to finally to 3:7, v/v to afford three subfractions (Fr.A-1 to Fr. A-3). Fr. A-1 (eluted with methanol/chloroform 1:9, v/v) was further subjected to medium pressure liquid chromatography (MPLC) with RP-C18 flash column (4 g) and eluted with methanol/water (MeOH/H_2_O) gradient to yield compound **1** (eluted with 7% MeOH/H_2_O, 2 mg) and compound **8** (eluted with 40% MeOH/ H_2_O, 2.4 mg). Fr. A-2 (eluted with chloroform/methanol 8.5:1.5, v/v) was purified by preparative TLC on silica plated with dichloromethane-MeOH- H_2_O (8:2:0.2) to yield compound **2** (9 mg). On Sephadex LH-20, Fr. A-3 (chloroform/methanol 8:2, v/v) was purified and 50% MeOH/ H_2_O was used as elution system to afford compound **3** (7.8 mg). Fraction B (150 mg) was purified using MPLC with RP-C18 flash column (12 g) and eluted with MeOH/ H_2_O gradient to yield compound **4** (eluted with 20% MeOH/ H_2_O, 8.0 mg), compound **5** (eluted with 25% MeOH/ H_2_O, 5.8 mg), compound **6** (eluted with 30% MeOH/ H_2_O, 20.2 mg) and compound **7** (eluted with 40% MeOH/ H_2_O, 7.5 mg). Direct comparison with standard compounds available in our laboratory and/or comparison with literature data were used for the identification of all the isolated compounds. Figure 1 shows the design of extraction and isolation of compounds of iridoids-rich fraction from *Pentas lanceolata* leaves (PIRF).

6β,7β-Epoxy-8-*epi*-splendoside (**1**): ^1^H NMR (600 MHz, CD_3_OD), δ 7.48 (d, *J* = 1.5 Hz, H-3, 1H), 5.77 (1H, s, H–l), 4.57 (d, *J* = 7.9 Hz, H–l", 1H), 3.88 (*dd*, *J* = 12.0, 2.0 Hz, H-6"a, 1H), 3.81 (d, *J* = 2.6 Hz, H-6, 1H), 3.74 (*s*, -COOMe, 3H), 3.68 (d, *J* = 11.7 Hz, H-10a, 1H), 3.65 (dd, *J* = 12.0, 5.9 Hz, H-6"b,, 1H), 3.51 (d, *J* = 2.6 Hz, H-7, 1H), 3.48 (d,* J* = 11.7 Hz, H-10b, 1H), 3.33–3.23 (H-5", H-4", and, H-3", overlapped with solvent signal, H-5), 3.13 (dd, *J* = 9.0, 8.1 Hz, H-2", 1H), 2.33 (d, *J* = 8.8 Hz, H-9, 1H); ^13^C NMR (150 MHz, CD_3_OD), δ 33.2 (C-5), 46.4 (C-9), 51.8 (COOMe), 57.9 (C-6), 60.8 (C-7), 62.8 (C-6"), 65.2 (C-10), 71.6 (C-4"), 74.5 (C-2"), 77.9 (C-5"), 78.4 (C-3"), 80.6 (C-8), 93.8 (C-1), 99.8 (C-1"),107.7 (C-4), 154.2 (C-3), 168.4 (C-11); ESI- MS: *m/z* 443.11 [M + Na].

Asperulosidic acid (**2**): ^1^H NMR (600 MHz, CD_3_OD), δ 7.41 (d, *J* = 1.4 Hz, H-3, 1H), 5.98 (d, *J* = 1.5 Hz, H-7, 1H), 4.97 (d,* J* = 8.9 Hz, H-1, 1H), 4.94 (d, *J* = 15.0 Hz, H-10a, 1H), 4.90 (dd, *J* = 6.0, 1.6 Hz, H-6, 1H), 4.81 (d,* J* = 15.0 Hz, H-10b, 1H), 4.72 (d, *J* = 7.9 Hz, H–l'', 1H), 3.83 (dd, *J* = 12.0, 1.5 Hz, H-6"a, 1H), 3.61 (m, H-6"b, 1H), 3.23–3.38 (H-2", H-3", H-4", and H-5", overlapped with solvent signal), 3.05 (t, *J* = 6.9 Hz, H-5, 1H), 2.58 (t, *J* = 8.1 Hz, H-9, 1H), 2.09 (s, CH_3_CO, 3H); ^13^C NMR (150 MHz, CD_3_OD), δ 20.7 (CO-Me), 43.8 (C-5), 46.9 (C-9), 63.0 (C-6"), 64.0 (C-10), 71.6 (C-4"),75.1 (C-2"),76.1 (C-6), 77.8 (C-5"),78.5 (C-3"), 100.5 (C-1"), 100.8 (C-1), 107.4 (C-4), 131.7 (C-7), 146.1 (C-8), 151.4 (C-3), 170.3 (C-11), 172.6 (CO-Me); ESI–MS: *m/z* 455.05 [M + Na]^+^.

Deacetyl-asperulosidic acid (**3**): ^1^H NMR, (600 MHz, CD_3_OD), δ 7.61 (s, H-3, 1H), 6.02 (d, *J* = 1.5 Hz, H-7, 1H), 5.05 (d, *J* = 8.9 Hz, H-1, 1H), 4.82 (H-6, 1H), 4.72 (d, *J* = 7.9 Hz, H–l", 1H), 4.46 (dd, *J* = 15.5, 1.1 Hz, H-10a, 1H), 4.22 (d, *J* = 15.5 Hz, H-10b, 1H), 3.85 (dd, J = 12.2, 1.8 Hz, H-6"a, 1H), 3.62 (dd, *J* = 12.0, 5.8 Hz, H-6"b, 1H), 3.39 (t, *J* = 8.8, H-5", 1H), 3.22–3.29 (3H, m, H-2", H-3"and H-4", 3H), 3.02 (t, *J* = 6.6 Hz, H-5, 1H), 2.56 (t, *J* = 7.9 Hz, H-9, 1H); ^13^C NMR (150 MHz, CD_3_OD), δ 172.0 (C-11), 154.6 (C-3), 151.5 (C-8), 129.8 (C-7), 109.6 (C-4), 101.4 (C-1), 100.4 (C-1"), 78.5 (C-3"), 77.8 (C-5"), 75.6 (C-6), 75.0 (C-2"), 71.7 (C-4"), 62.9 (C-6"), 61.7 (C-10), 46.0 (C-9), 43.0 (C-5). ESI–MS: *m/z* 413.28 [M + Na]^+^.

13R-*epi*-Epoxygaertneroside (**4**): ^1^H NMR (600 MHz, CD_3_OD) δ 7.55 (d, *J* = 1.6 Hz, H-3, 1H), 7.22 (d, *J* = 8.6 Hz, H-2', H-6', 2H), 7.04 (d, *J* = 1.5 Hz, H-10, 1H), 6.77 (d, *J* = 8.6 Hz, H-3', H-5', 2H), 5.40 (d,* J* = 1.2 Hz, H-13, 1H), 5.33 (brs, H-1, 1H), 4.54 (d, *J* = 7.9 Hz, H-1", 1H), 4.03 (d, *J* = 2.5 Hz, H-7, 1H), 3.85 (dd, *J* = 11.8, 1.2 Hz, H-6"a, 1H), 3.77 (s, -COOMe, 3H), 3.70 ( m, H-6"b, 1H), 3.46 (d, *J* = 7.6 Hz, H-5, 1H), 3.35 (d, *J* = 2.5 Hz, H-6, 1H), 3.12–3.35 (H-2", H-3", H-4", and H-5", overlapped with solvent signal), 2.77 ( dd,* J* = 8.3, 1.1 Hz, H-9, 1H); ^13^C NMR (150 MHz, CD_3_OD), δ 33.0 (C-5), 43.6 (C-9), 51.9 (COOMe), 57.8 (C-7), 59.2 (C-6), 62.5 (C-6"), 69.6 (C-13), 71.3 (C-4"), 74.4 (C-2"), 77.8 (C-5"),78.4 (C-3"),92.6 (C-8), 92.8 (C-1), 99.6 (C-1"), 108.0 (C-4), 116.3 (C-3', C-5'), 129.3 (C-2', C-6'), 133.0 (C-1'), 140.5 (C-11), 148.0 (C-10), 153.9 (C-3), 158.6 (C-4'), 168.0 (C-14), 171.7 (C-12); ESI- MS: *m/z* 587.13 [M + Na].

Gaertneroside (**5**): ^1^H NMR (600 MHz, CD_3_OD) δ 7.51 (d, *J* = 1.6 Hz, H-3, 1H), 7.45 (d, *J* = 1.3 Hz, H-10, 1H), 7.28 (d, *J* = 8.5 Hz, H-2', H-6', 2H), 6.78 (d, *J* = 8.5 Hz, H-3', H-5', 2H), 6.47 (dd, *J* = 5.6, 2.5 Hz, H-6, 1H), 5.56 ( dd, *J* = 5.6, 2.2 Hz, H-7, 1H), 5.36 (d, *J* = 1.2 Hz, H-13, 1H), 5.15 (d, *J* = 4.9 Hz, H-1, 1H), 4.67 (d, *J* = 7.9 Hz, H-1", 1H), 3.91 (m, H-5, 1H), 3.79 (dd, *J* = 12.1, 2.2 Hz, H-6"a, 1H), 3.75 (s, -COOMe, 3H), 3.69 (m, H-6"b, 1H), 3.21–3.38 (H-2", H-3", H-4", and H-5", overlapped with solvent signal), 2.90 (dd, *J* = 7.6, 4.9 Hz, H-9, 1H); ^13^C NMR (150 MHz, CD_3_OD), δ 40.3 (C-5), 50.8 (C-9), 51.9 (COOMe), 62.2 (C-6"),69.9 (C-13), 70.9 (C-4"),74.4 (C-2"),77.8 (C-5"),78.4 (C-3"), 94.4 (C-1), 98.0 (C-8), 100.5 (C-1"),110.9 (C-4), 116.3 (C-3'/C-5'), 129.6 (C-2'/ C-6'), 129.9 (C-7), 133.2 (C-1'), 137.9 (C-11), 141.6 (C-6), 150.1 (C-10), 152.5 (C-3), 158.6 (C-4'), 168.4 (C-14), 172.4 (C-12); ESI- MS: *m/z* 547.14 [M-H]^−^.

13R-*epi*-Gaertneroside (**6**): ^1^H NMR (600 MHz, CD_3_OD) δ 7.48 (d, *J* = 1.5 Hz, H-3, 1H), 7.25 (d,* J* = 1.3 Hz, H-10, 1H), 7.21 (d, *J* = 8.6 Hz, H-2', H-6', 2H), 6.77 (d, *J* = 8.6 Hz, H-3', H-5', 2H), 6.45 (dd, *J* = 5.6, 2.6 Hz, H-6), 5.47 (dd, *J* = 5.6, 1.8 Hz, H-7, 1H), 5.40 (d, *J* = 1.0 Hz, H-13, 1H), 5.35 (d, *J* = 3.8 Hz, H-1, 1H), 4.65 (d, *J* = 7.9 Hz, H-1", 1H), 3.90 (m, H-5, 1H), 3.88 (dd, *J* = 11.8, 1.0 Hz, H-6"a, 1H), 3.74 (s, -COOMe, 3H), 3.65 (m, H-6"b, 1H), 3.36 ( m, H-3", 1H), 3.28–3.30 (H-4", and H-5", overlapped with solvent signal), 3.22 (dd, *J* = 9.2, 8.0 Hz, H-2", 1H), 3.00 (dd, *J* = 7.9, 3.9 Hz, H-9, 1H); ^13^C NMR (150 MHz, CD_3_OD), δ 39.9 (C-5), 50.6 (C-9), 51.9 (COOMe), 62.6 (C-6"), 69.5 (C-13), 71.4 (C-4"), 74.5 (C-2"), 77.8 (C-5"), 78.5 (C-3"), 93.8 (C-1), 97.9 (C-8), 99.8 (C-1"), 111.4 (C-4), 116.3 (C-3'/C-5'), 129.2 (C-2'/C-6'), 130.1 (C-7), 133.2 (C-1'), 137.9 (C-11), 141.0 (C-6), 150.5 (C-10), 152.2 (C-3), 158.5 (C-4'), 168.4 (C-14), 172.4 (C-12); ESI- MS: *m/z* 547.14 [M-H]^−^.

13R-Methoxy-*epi*-gaertneroside (**7**): ^1^H NMR (600 MHz, CD_3_OD) δ 7.49 (d, *J* = 1.3 Hz, H-3, 1H), 7.30 (brs, H-10, 1H), 7.19 (d, *J* = 8.5 Hz, H-2', H-6', 2H), 6.77 (d, *J* = 8.5 Hz, H-3', H-5', 2H), 6.45 ( dd, *J* = 5.6, 2.5 Hz, H-6, 1H), 5.45 (dd, *J* = 5.6, 1.9 Hz, H-7, 1H), 5.34 (d, *J* = 4.4 Hz, H-1, 1H), 4.95 (brs, H-13, 1H), 4.67 (d, *J* = 7.9 Hz, H-1", 1H), 3.90 (m, H-5, 1H), 3.86 (m, H-6"a, 1H), 3.75 (s, -COOMe, 3H), 3.65 (m, H-6"b, 1H), 3.27–3.39 (H-3", H-4",H-5" and -OCH_3_, overlapped with solvent signal), 3.22 (1H, d, *J* = 9.0 Hz, H-2", 1H), 2.97 (dd, *J* = 7.7, 4.4 Hz, H-9, 1H); ^13^C NMR (150 MHz, CD_3_OD), δ 40.2 (C-5), 50.7 (C-9), 51.9 (COOMe), 57.2 (-OCH_3_), 62.8 (C-6"), 71.5 (C-4"), 74.6 (C-2"), 77.8 (C-5"), 78.5 (C-3"), 78.9 (C-13), 93.9 (C-1), 98.0 (C-8), 99.8 (C-1"), 111.3 (C-4), 116.4 (C-3'/C-5'), 129.8 (C-2'/C-6'), 129.9 (C-7), 130.2 (C-1'), 135.8 (C-11), 141.3 (C-6), 150.9 (C-10), 152.4 (C-3), 158.9 (C-4'), 168.4 (C-14), 172.3 (C-12); ESI- MS: *m/z* 561.14 [M-H]^−^.

Kaempferol-3-*O*-robinobioside (**8**): ^1^H NMR (600 MHz, CD_3_OD) δ 8.09 (d, *J* = 8.8 Hz, H-2', 6', 2H), 6.88 (d, *J* = 8.8 Hz, H-3', 5', 2H), 6.38 (d, *J* = 1.8 Hz, H-6, 1H), 6.19 (d, *J* = 1.8 Hz, H-8, 1H), and 5.02 (d, *J* = 7.8, H-1", 1H), 4.52 (brs, H-1"', 1H), 1.18 (d, *J* = 6.3, H-6"', 3H). ^13^C NMR (150 MHz, CD_3_OD) δ 17.91 (C-6'''), 67.4 (C-6''), 69.7 (C-4''), 70.2 (C-5'''), 72.1 (C-3'''), 72.3 (C-2'''), 73.0 (C-2''), 73.9 (C-4"'), 75.1 (C-3"), 75.4 (C-5"), 95.3 (C-8), 100.5 (C-6), 101.9 (C-1"'), 105.1 (C-1"), 105.7 (C-10), 116.1 (C-3'/C-5'), 122.7 (C-1'), 132.4 (C-2'/C-6'), 135.7 (C-3), 158.7 (C-9), 159.1 (C-2), 161.6 (C-4'), 162.9 (C-5), 167.8 (C-7), 179.4 (C-4); ESI–MS *m/z*: 593.15 [M-H]^−^

### Experimental design of in vitro and in vivo studies

**Fig. 2:**
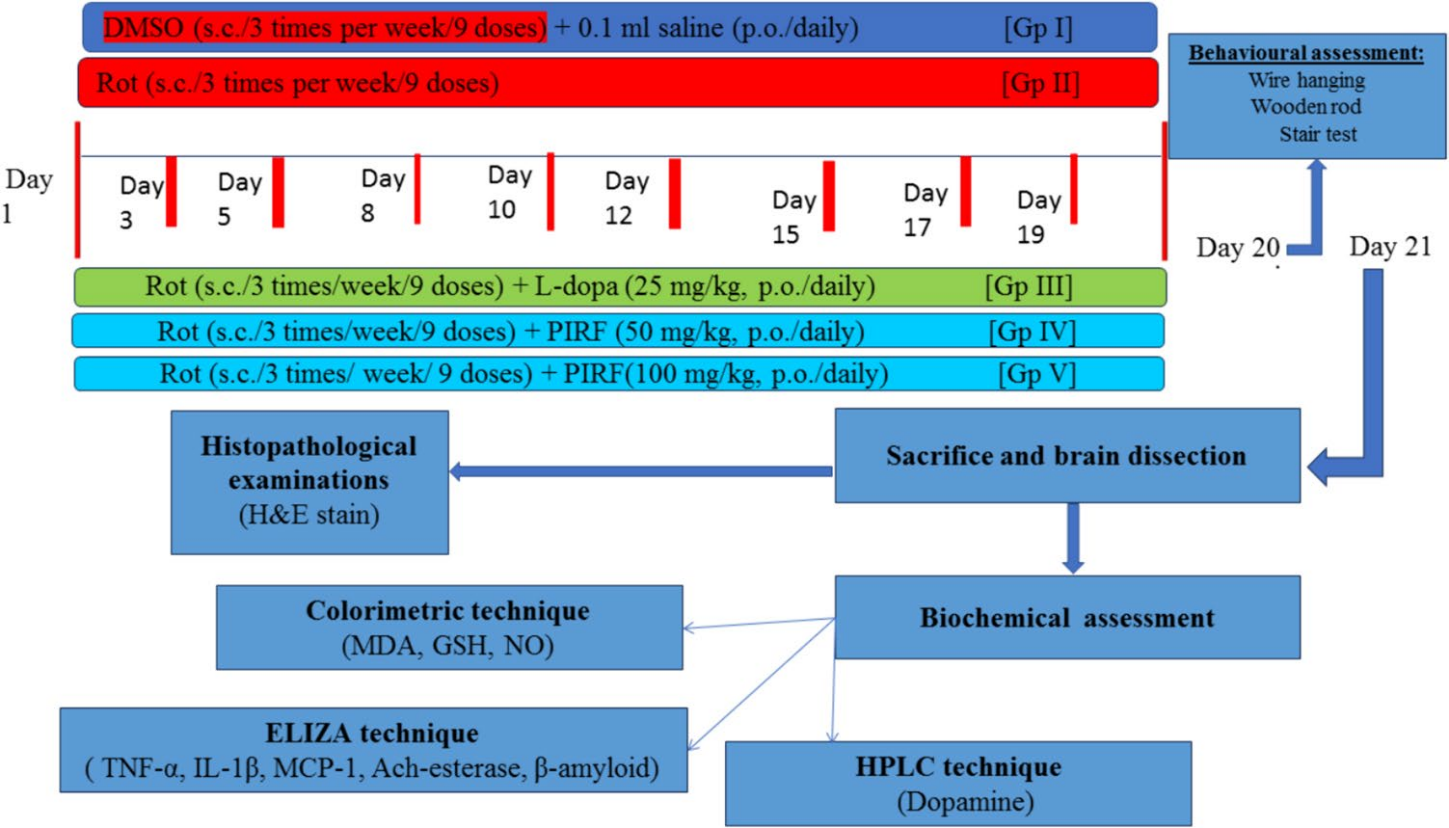
Experimental design of in vitro and in vivo anti-parkinsonian study. DMSO, Dimethylsulfoxide, PIRF, The iridoids-rich fraction isolated from *Pentas lanceolata* leaves, Rot, Rotenone

As shown in Fig. 2, the experimental design of in vitro and in vivo studies was carried out.

#### In vitro studies on BDNF and NGF

The human colonic epithelial cell line (Caco-2) and human astrocytoma cell (1321N1) were incubated and maintained in Dulbecco's Modified Eagle Medium with high glucose (DMEM) with 10% fetal bovine serum (FBS). At a density of 0.5 × 10^5^ cells/well, the cells were incubated in 24-well plate in a humidified atmosphere (at 37 °C) and containing 5% CO_2_.

At 24 h after seeding, cells were treated with each sample of PIRF dissolved in dimethyl sulfoxide (DMSO) solution. After 24 h, cells were collected, and then total RNA was extracted from the cultured cells using PureLink RNA Mini kit (Invitrogen, CA, USA). The cDNA strand was synthesized from 400 ng of total RNA using ReverTra Ace qPCR RT Master Mix (TOYOBO, Osaka, Japan). THUNDERBIRD SYBR qPCR Mix (TOYOBO, Osaka, Japan) was used for quantitative real-time PCR. Real-time PCR was performed using AriaMX Real-time PCR System (Agilent Technologies, CA, USA) under the following conditions: 3 min (at 95 °C), followed by 40 cycles for 3 s (each of 95 °C), for 30 s (60 °C). Primers used for amplification were as follows: GTCAAGTTGGGAGCCTGAAATAGTG and AGGATGCTGGTCCAAGTGGTG for BDNF, ACCTTTCTCAGTAGCGGCAA and TGTGTCACCTTGTCAGGGAA for NGF and GGGTCAGAAGGACTCCTATG and GTAACAATGCCATGTTCAAT for β-actin as an internal control. PCR products were analyzed using AriaMx Real-time PCR System Software.

#### Experimental design of the in vivo study

We have five groups with eleven male mice in each one: Control group receiving vehicle (DMSO), induced group using rotenone (1.5 mg/kg, three times a week), the last three groups received rotenone together with one of the following oral treatments: L-Dopa (reference drug, 25 mg/kg), 50 mg /kg PIRF, 100 mg /kg PIRF.

### Animals

Male mice were divided into five groups. They were housed with free access to standard chow diet and water at humidity (60 ± 10%), constant temperature (25 ± 2 °C), and a 12/12-h light/dark cycle. According to the guidelines for the Care and Use of Laboratory Animals published by the US National Institutes of Health (NIH Publication No. 85–23, revised 2011), the investigation was carried out. It was performed in agreement with ethical procedures approved by the Ethics Committee of Safety and Health Committee in NRC (Ethics number: 04420124).

### Methodology

#### Induction of PD

The method of Abdel-Salam et al. (2014) was used for rotenone-induced PD. Rotenone (dose of 1.5 mg/kg/day) was given as a solution after dissolving in DMSO, via the S.C. route (for 3 weeks, three times/ week) to make a total of nine doses. Starting from the first day of rotenone, the treatment with L-dopa or PIRF (50 or 100 mg/kg) will be given orally daily for 21 consecutive days.

#### Behavioral study

Twenty-four hours after the last treatment day, three behavioral tests were accomplished to assess motor functions in mice, specifically stair test, wood-walking test, and wire-hanging test (El-Shamarka et al. 2020).

#### Stair test

The stair test aims to assess skilled motor coordination. Briefly, at the bottom of a wooden stair, each mouse was added. From the experimental bench, they were placed at an angle of 55 °C. For each mouse, the time spent climbing the stairs three times was recorded.

#### Wood-walking test

The mice were allowed to walk along a wooden stick (1 m long and 1 cm thick). For each mouse, the time spent reaching the end of the stick is recorded to assess motor coordination (three trials for each mouse).

#### Wire-hanging test

It is also called the horizontal bar test. It is done by hanging the mice from a steel rod by their forelimbs, placed 25 cm above the bench. The rod was 0.2 cm in diameter and 25 cm long. For each mouse, the time could stay hanging itself suspended from the rod is the latency time. For three trials, the latency time (with a cut-off time of 60 s) was recorded.

#### Tissue sampling

Using urethane, the mice were anesthetized at the end of behavioral tests and were killed by cervical dislocation. The right and left striata are detached directly after rapid separation of the brains. They were kept on ice and kept at − 80 °C for further handling. In each group, five brains are collected from 5 mice. A homogenizer (yellow line, DI18 basic, Germany) was used to homogenize the right striatum in phosphate buffered saline (PBS; 0.1 M, pH = 7.4) to prepare 20% homogenate. A cooling centrifuge (Sigma 3–30 k, USA) was used to centrifuge the homogenate (1000 *xg* for 15 min at 4 °C). The supernatant was kept at − 80 °C for further colorimetric assay of antioxidants. In ice-cold saline, the left striatum is homogenized to prepare 25% *w*/*v* homogenate of each mouse. The homogenate was centrifuged at 4 °C for 20 min. at 1000 *xg* and stored at − 80 °C for additional ELISA of TNF-α, IL-1β, Ach E, β- amyloid and MCP-1. Then three brains from each group were homogenized in methanol (HPLC grade), then centrifuged and the supernatant was kept at -80 for determination of dopamine level using HPLC technique. Alternatively, in well-sealed containers, the rest three brains are separately immersed in formalin solution (10%) in normal saline and kept for histopathological investigation after tissue hardening.

### Biochemical parameters

#### Determination of reduced glutathione (GSH content)

Briefly, Ellman´s reagent or 5,5'-dithiobis (2-nitrobenzoic acid) (DTNB) is reduced by the free sulfhydryl group on GSH molecule to generate 5- thio-2-nitrobenzoic acid which has yellow color and can be determined by reading absorbance at 412 nm (Ellman 1959).

#### Lipid peroxidation determination

The product of lipid peroxidation; malondialdehyde (MDA) was measured according to the method of Nair and Turner (1984). The absorbance (at λ532 nm) was recorded using a spectrophotometer for the product of thiobarbituric acid-forming TBA-MDA adducts reacted with thiobarbituric acid reactive substances (TBAS).

#### Nitric oxide content determination

Griess reagent was used for measuring the nitric oxide (NO) content (Moshage et al. 1995). By nitrate reductase, the nitrate is converted to nitrite. Nitrite is converted to a deep purple azo compound by Griess reagent. The absorbance was measured at by a spectrophotometer (at λ 540 nm). As an indicator to produce nitric oxide, nitrite was mostly used as a stable end-product of nitric oxide radical.

#### ELISA of TNF-α, IL-1 β, AchE, β- amyloid and MCP-1

Estimation of striatal TNF-α, IL-1, AchE, β- amyloid and MCP-1 were performed using ELISA technique by test kits obtained from Sunlong Biotech Co. LTD with the following catalogue number: SL0722Ra, SL0402RA, SL0027Ra, SL1392Ra, SL0497Ra according to manufacturer instructions based on the sandwich principle by the aid of ELISA reader (Model Spectra Max Plus-384 Absorbance Microplate Reader, USA).

#### Determination of dopamine content

Dopamine was analyzed in brain supernatant using Dionex Ultimate 3000 UHPLC (Thermo Scientific, USA). The separation was carried out on Zorbax Eclipse XDB C_18_ (4.6X150 mm, 5 µm). The mobile phase was phosphate buffer (pH, 3.0): methanol (80:20, v/v) at flow rate 0.5 ml/min. The detection wavelength was 270 nm.

#### Histopathological study

Tissues are hardened for at least 7 days. The sections of striata were stained after preparation according to method of Bancroft and Steven (1983). For 1 h, these sections were washed with water. The hardened sections were dehydrated in graded concentrations of ethanol. In melted paraffin wax, the cleared specimens (in xylene) are then embedded. Drying (for 4–6 h) of sample was carried out in an oven at 70 °C. The sections of tissue (3–5 μm thick) were prepared by rotary microtome. The routine hematoxylin and eosin (H and E) stain was used for staining.

### Statistical analysis

The statistical analysis was done using one-way analysis of variance (ANOVA) test. Data are expressed as means ± standard error of the mean (SEM). ANOVA test was followed by Tukey–Kramer post hoc test to compare all means pairwise. This was accomplished by the aid of statistical package for social sciences computer software version 22 (SPSS Inc., Chicago, USA). A *P*-value less than 0.05 is typically considered to be statistically significant.

## Results

### Chemical structures of isolated compounds (1-8) from *Pentas lanceolata* leaves

Chromatographic fractionation and purification of *Pentas lanceolata* iridoids rich fraction PIRF afforded seven iridoid glycosides and one flavonoid diglycoside (Fig. 1).

**Fig. 3:**
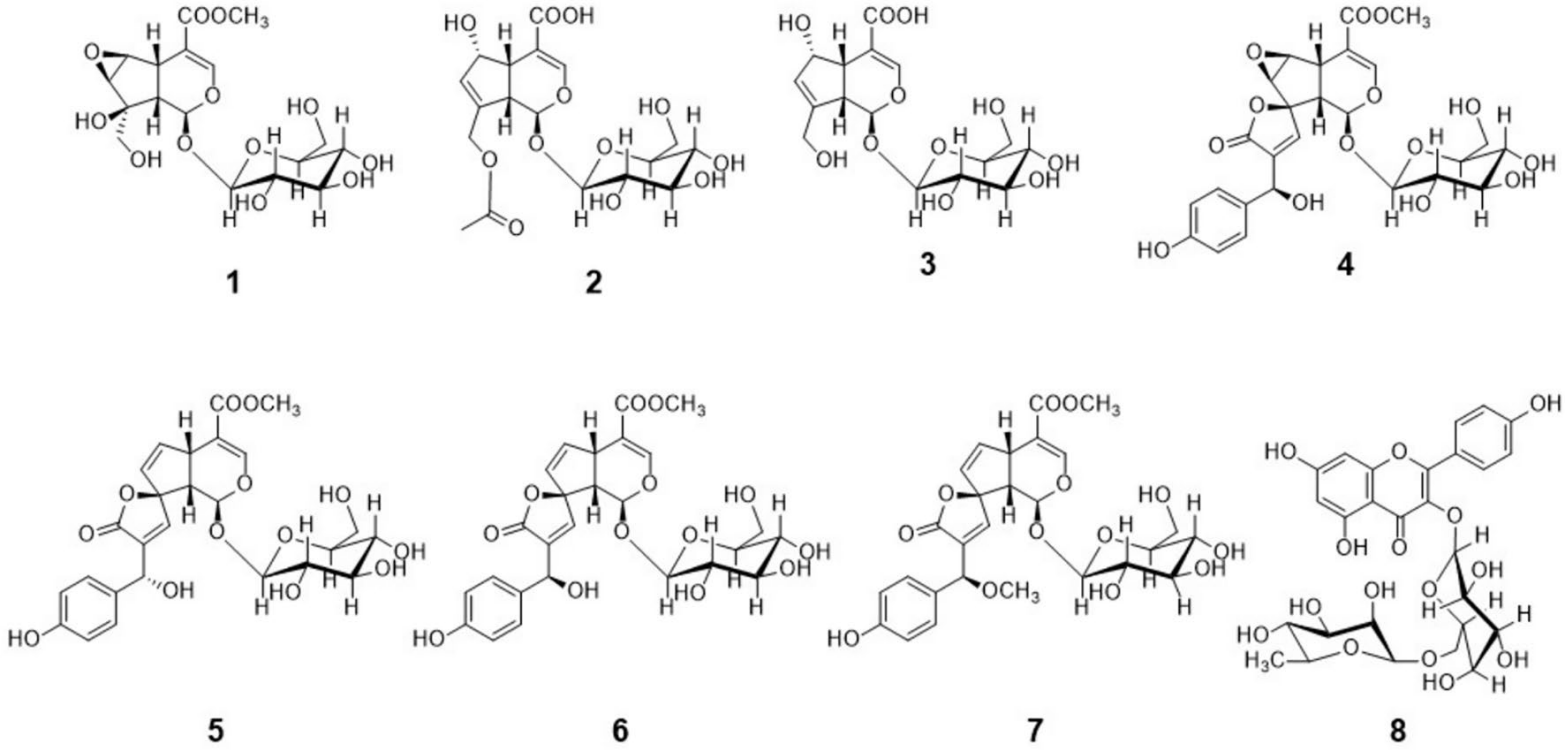
Chemical structures of isolated compounds **1**–**8** from *Pentas lanceolata* iridoids-rich fraction

Inspection of the ^1^H and ^13^C NMR data of compounds **1**–**7** (in CD_3_OD) revealed the presence of characteristic resonances of iridoid glycoside phytochemical class by comparison with the reported literature. The compounds were 6β,7β-epoxy-8-*epi*-splendoside (**1**) (Su et al. 2005), asperulosidic acid (**2**) (Demirezer et al. 2006); deacetyl-asperulosidic acid (**3**) (Demirezer et al. 2006); 13*R*-*epi*-epoxygaertneroside (**4**) (Schripsema et al. 2007); gaertneroside (**5**); (Cimanga et al. 2003); 13*R*-*epi*-gaertneroside (**6**) (Schripsema et al. 2007); 13*R*-methoxy-*epi*-gaertneroside (**7**) (Abd-Alla et al. 2017); kaempferol-3-*O*-robinobioside (**8**) (Yasukawa and Takido 1987) (Fig. 3).

The structure of compounds **1**–**7** as iridoid glycosides were illustrated through the observation of ^1^H NMR signals at δ_H_ 7.41–7.61 (1H, d, *J* $\approx$ 1.5, H-3), 4.97–5.77 (1H, H-1), 4.54–4.72 (1H, d, *J* = 7.9), 3.02–3.91 (1H, H-5) and 2.33–3.00 (1H, H-9) and ^13^C NMR signals at δ_C_ 168.4–170.3 (C-11), 152.2–154.2 (C-3), 107.7–111.4 (C-4), 99.8–100.5 (Glc-1) and 92.8–101.4 (C-1). Compound **1** showed molecular ion peak at *m/z* 443.11 [M + Na] in ESI–MS analysis. Beside the iridoid glycoside nucleus characteristic signals, NMR spectra of compound** 1** showed two coupled oxygenated protons at δ_H_ 3.51 (d, *J* = 2.6, 1H) and 3.81 (d, *J* = 2.6, 1H) and three oxygenated carbons at δ_C_ 57.9, 60.8 and 80.6 suggesting the occurrence of epoxy group between C-7 and C-6 and hydroxyl group at C-8. The data of ^1^H and ^13^C NMR of compound **1** was identical to that of 6β,7β-epoxy-8-*epi*-splendoside. Compounds **2** and **3** showed molecular ion peaks at *m/z* 455.05 [M + Na]^+^ and 413.28 [M + Na]^+^ respectively in ESI–MS analysis. The NMR spectra of the two compounds were very similar except for the appearance of methyl protons resonance at δ_H_ 2.09 (3H, s), methyl carbon signal at δ_C_ 20.7 and carbonyl signal at δ_C_ 172.6. These findings beside the 42 Da mass differences between the two compounds suggested that compound **3** is a deacetylated derivative of compound **2**. The spectral data of ^1^H and ^13^C NMR of compounds **2** and **3** were identical to that of Asperulosidic acid and deacetyl-asperulosidic acid, respectively. Compounds **4**–**7** showed ^1^H NMR signals at δ_H_ 7.19–7.28 (d, *J* = 8.5, 2H), 6.77 (d, *J* = 8.5, 2H) and ^13^C NMR signals at δ_C_ 129.2–129.9, 116.3, 171.7–172.4, 148.0–151.1, 135.8–140.5 and 98.0 indicating the occurrence of *para*-substituted phenyl ring and spirolactone functionality. Compounds **4** showed molecular ion peaks at *m/z* 587.13 corresponding for [M + Na] + adduct. The presence of 6, 7-epoxide was indicated by ^1^H NMR signals at δ_H_ 3.35 (d, *J* = 2.6, 1H) and 4.03 (d, *J* = 2.6, 1H) and ^13^C NMR signals at δ_C_ 57.8 and 59.2. By comparison of compound **4** NMR data with literature it was identified as 13R-*epi*-epoxygaertneroside. Both compounds **5** and **6** revealed molecular ion peaks at *m/z* 547.14 [M-H]^−^. Their NMR spectral data (^1^H and ^13^C) were similar to that of compound **4** except for the disappearance of resonances corresponding for 6,7 -epoxide and appearance of ^1^H NMR signals at δ_H_ 6.47 (dd, *J* = 5.6, 2.5, 1H) and 5.56 (dd, *J* = 5.6, 2.2, 1H) for compound **5** and at δ_H_ 6.45 (dd, *J* = 5.6, 2.6, 1H) and 5.47 (dd, *J* = 5.6, 1.8, 1H) for compound **6**, in addition to ^13^C NMR signals at δ_C_ 141.6 and 129.9 for compound **5** and δ_C_ 141.0 and 130.1 for compound **6** suggesting replacement of 6,7 -epoxide in compound **4** with 6,7- double bond. Moreover, NMR spectra of compounds **5** and **6** were almost identical except for the ^1^H NMR signal at δ_H_ 5.15 in compound **5** that appeared more downfield at δ_H_ 5.35 in compound **6** and the signal at δ_H_ 7.45 in compound **5** that appeared upfield at δ_H_ 7.25 in compound **6**. NMR data for compounds **5** and **6** were identical to that of gaertneroside and13R-*epi*-gaertneroside_,_ respectively. Compound **7** showed molecular ion peaks at *m/z* 561.14 [M-H]^−^. NMR spectral data (^1^H and ^13^C) for compound **7** were very similar to compound **6** except for the appearance of methoxy signal at δ_H_ 3.31 (3H, s) and at δ_C_ 57.2. Also, downfield shift of signal at δ_H_ 7.25 in compound **6** to δ_H_ 7.30 and upfield shift of signal at δ_H_ 5.40 in compound **6** δ_H_ 4.95 were also observed. ^1^H and ^13^C NMR data for compound **7** came identical with 13R-methoxy-*epi*-gaertneroside.

Compound **8** showed showed molecular ion peaks at *m/z* 593.15 [M-H]^−^ in ESI–MS. NMR spectral data (^1^H and ^13^C) for compound **8** in CD_3_OD revealed characteristic signals for 3-*O-*substituted kaempferol structure. Also, two signals of anomeric protons at δ_H_ 5.02 (d, *J* = 7.8, 1H) and 4.52 (brs, 1H) and carbons at δ_C_ 105.1 and 101.9 and methyl signal at δ_H_ 1.18 (3H, s) and δ_C_ 17.91. By comparison of compound **8** NMR data with literature it was identified as the common flavonoid, kaempferol-3-*O*-robinobioside. Supplementary data (Supporting Figures S1–S16) of the chromatogram of compounds **1**–**8** are available to this article.

### In vitro studies on BDNF and NGF

**Fig. 4:**
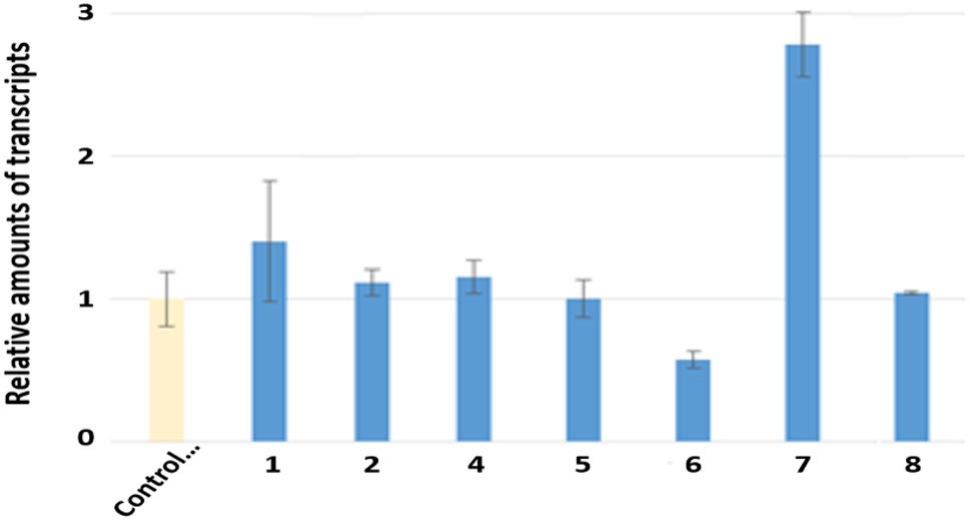
The expression of BDNF gene in human colonic epithelial cell line (Caco-2)

**Fig. 5:**
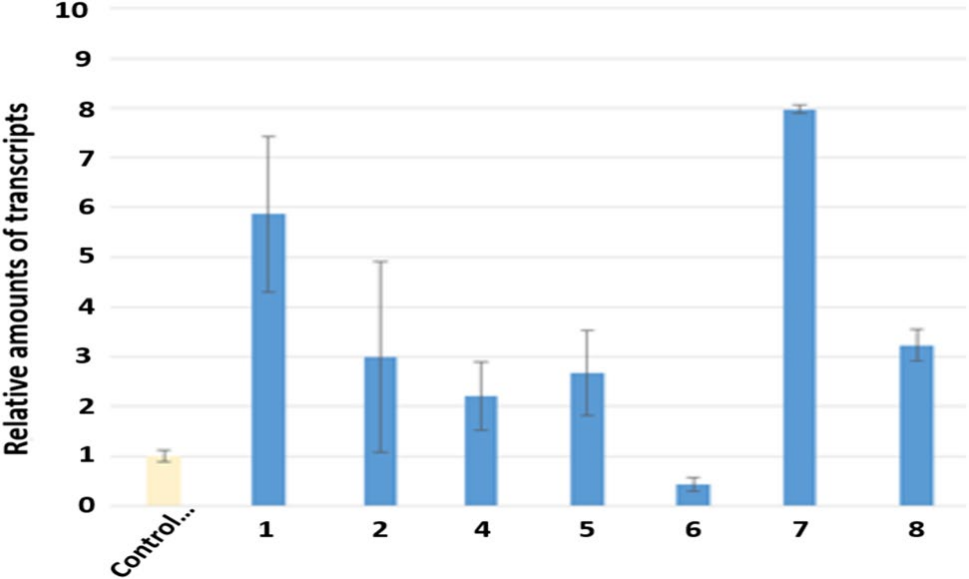
The expression of NGF gene in human astrocytoma cell (1321N1)

In Caco-2 cells, only compound **7** significantly induced the expression of BDNF gene (Fig. 4). On the other hand, the expression of NGF genes was significantly increased by compounds **1**, **2**, **4**, **5**, **7** and **8** (Fig. 5). Especially, compound **7** significantly induced both genes expression. In result, compound **7** related BDNF production in Caco-2 cells and then some compounds are involved in NGF production in 1321N1 cells. The result indicates that these compounds have the possibility of anti-Parkinson’s disease.

### Behavioral results

Treatment of mice with rotenone for induction of Parkinsonism had been justified by the behavioral tests for measurement of grip strength (wire-hanging test) and motor coordination (stair test and wooden-walking test). In the wire-hanging test as seen in Fig. 4A, treatment of mice with rotenone decreased the wire-hanging time by about 81% in comparison with control mice.

**Fig. 6:**
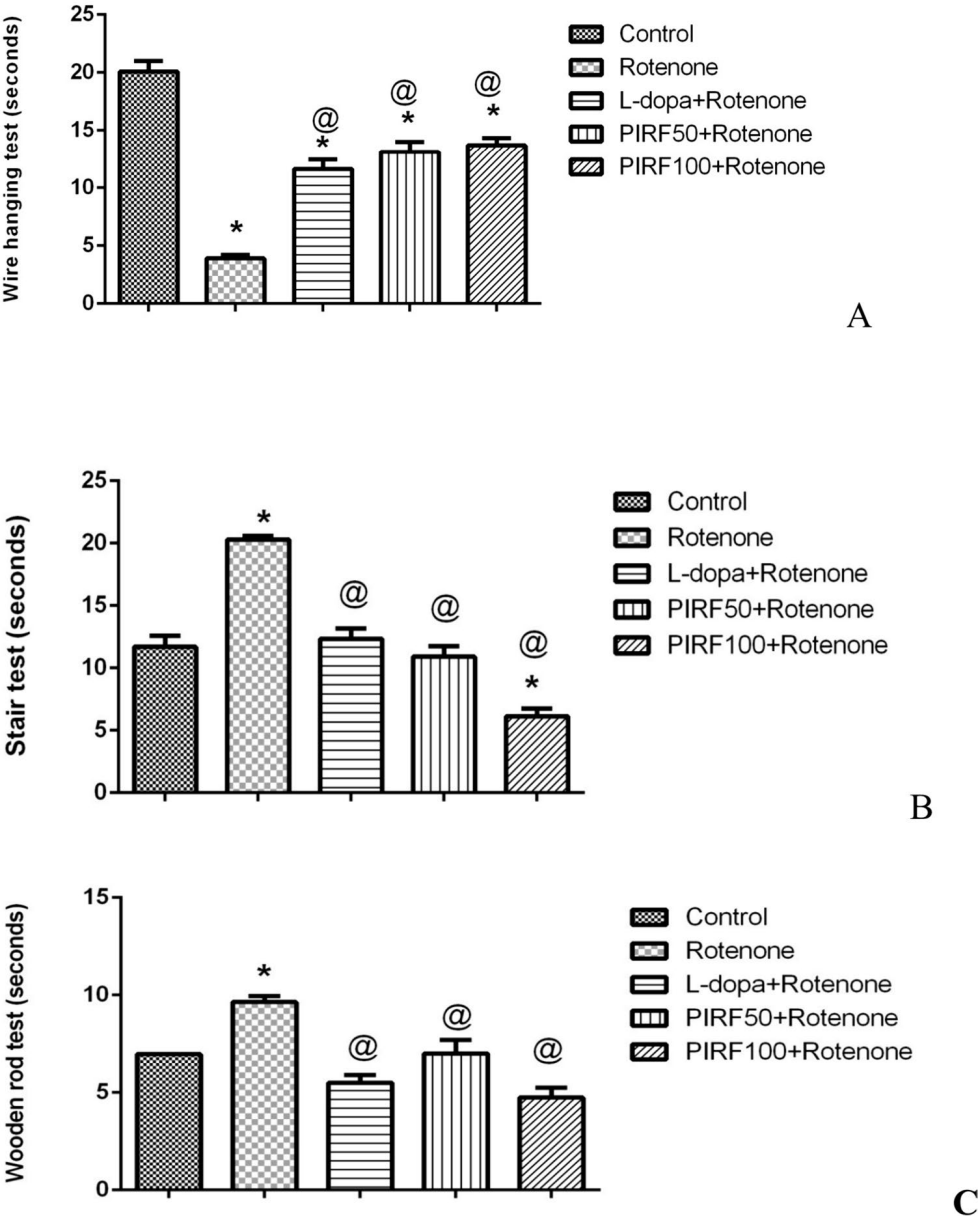
Effect of PIRF treatment in rotenone model of Parkinsonism on behavioral tests. **A,** In the wire-hanging test. **B,** In the stair test. **C,** In the wooden-walking test. *, means significantly different from control group; @, significantly different from rotenone group. Each value represents the mean of 11 measurements ± standard error of mean (SEM)

Our previous study (Arafa et al. 2009) reported the in vivo anti-amnesic effect of PIRF against D-galactose induced brain aging with 50 mg/kg, as a selected dose. In the current study, we investigate the effect of the double dose (100 mg/kg) as well as the previous dose (50 mg/kg) for the design of experiment. This was the way to choose the appropriate dose that exerts the anti-PD activity with a lower dose of choice. The grip strength was improved by treatment with either L-dopa (189%) or PIRF 50 and 100 mg/kg (235 and 250.4% respectively) as compared with rotenone group although this improvement didn’t reach the normal range. In Fig. 6B, treatment with rotenone increased the time taken by mice to climb up the stairs by 73.5% when compared with time taken by control mice. Mice in L-dopa group reached the end of the stairs in the same time as that of control mice. Mice in the 2 groups of PIRF were even much better in the stair test as they consumed less time to climb the stairs when compared with the control group. In Fig. 6C rotenone treated mice consumed more time (38.3%) to reach the end of the wooden-walking as compared with control group. Mice treated with either L-dopa or PIRF 100 mg/kg were even better than control mice in walking on the wooden walking. From the previous behavior results we deduce that treatment with PIRF had counteracted the effect of rotenone on grip strength and motor coordination as they were restored back to normal levels.

### Biochemical results

The loss of dopaminergic neurons in PD is often in parallel by changes in activation status and the numbers of astrocytes, microglia and adaptive and innate immune cells. Increasing the anti-inflammatory M2 phenotype microglia by inhibiting the proinflammatory M1 polarization may provide a solution for the treatment of PD (Jin et al. 2023).

**Fig. 7:**
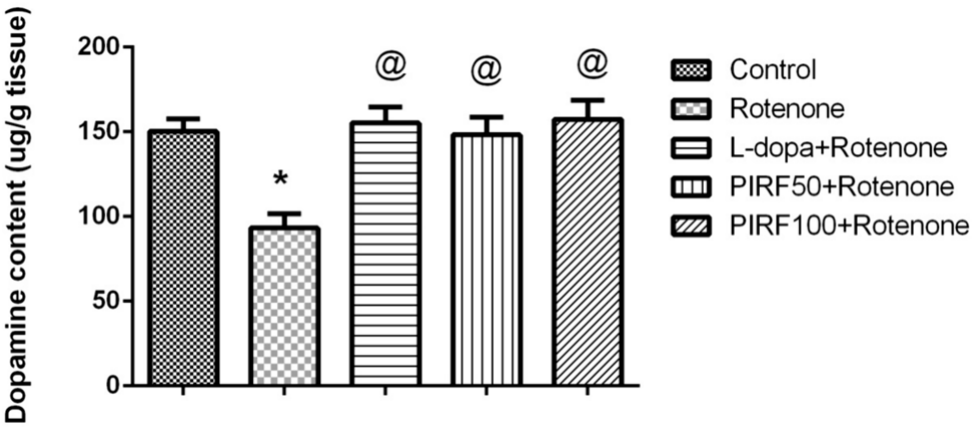
Effect of PIRF treatment in rotenone model of Parkinsonism on dopamine level. *, means significantly different from control group. Each value represents the mean of 8 values ± standard error of mean (SEM)

Figure 7a showed that treatment with rotenone had caused depletion of dopamine content in brain homogenate by 38% when compared with the control mice. Treatment with either L-dopa or PIRF (both doses) had improved the dopamine content as it reached the normal level as in control mice.

In the current study, kaempferol-3-*O*-robinobioside (compound **8**) is a flavonoid diglycoside of kaempferol isolated from PIRF. Kaempferol was reported directly inhibits Aβ deposition in Alzheimer disease and α-synuclein aggregation and Lewy body formation in PD. Kaempferol promotes dopamine release in the brain and improves motor dysfunction in PD. kaempferol may directly regulated striatal dopamine levels and improved motor symptoms in animal models (Cai et al. 2022; Jin et al. 2023).

**Fig. 8:**
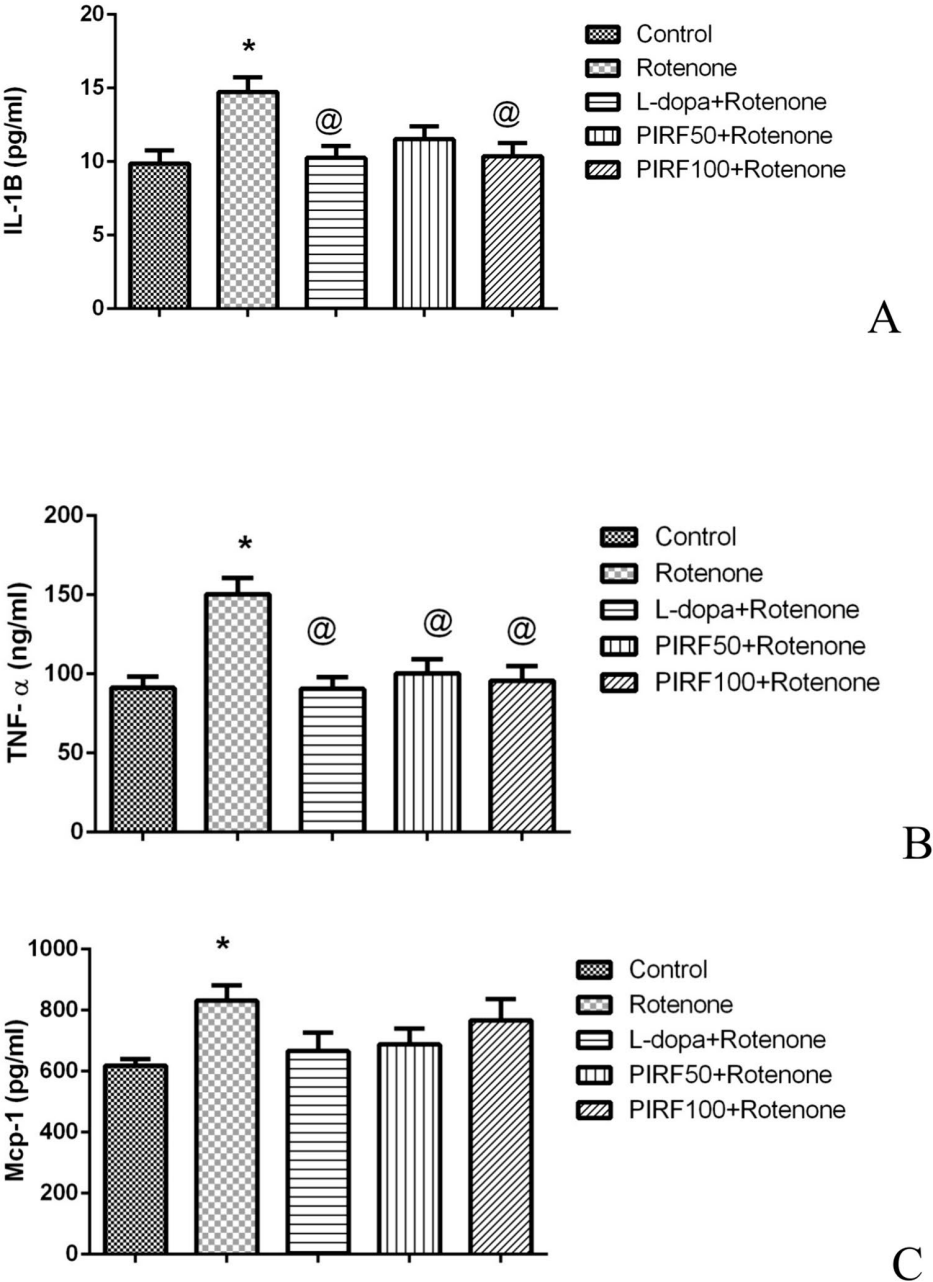
Effect of PIRF treatment in rotenone model of Parkinsonism on inflammatory markers. **A** on IL-1β content, **B** on TNF- α content, and **C** on monocyte chemotactic protein-1 content. *, significantly different from control group. Each value represents the mean of 8 values ± standard error of mean (SEM)

Figure 8A, B showed the inflammatory cytokines interleukin-1β and tumor necrosis factor-α contents that were elevated by 49 and 64.7% respectively due to treatment with rotenone when compared with their content in control mice. Treatment with either L-dopa or PIRF (both doses) masked the inflammatory effect of rotenone as the IL-1β and TNF- α returned to their normal content as in the control group. In Fig. 8C the proinflammatory cytokine MCP-1that was elevated by 34.3% due to treatment with rotenone restore its content to normal due to treatment with L-dopa and PIRF (both doses).

In the present study, kaempferol-3-*O*-robinobioside (compound **8**) is a flavonoid diglycoside of kaempferol isolated from PIRF. Flavonoids decreased the release of inflammatory mediators as well as inhibited macrophage/monocytes, mast cells and T cells (Abd-Alla et al. 2021, 2022). In PD rats, kaempferol has been reported to reduce the loss of tyrosine hydroxylase (TH)-positive neurons, improve motor dysfunction, inhibit microglial activation, and downregulate the levels of inflammatory factors IL-18 and IL-1β (Cai et al. 2022; Jin et al. 2023). The activities of the compounds and its derivatives as anti-apoptotic and anti-oxidant activities play important roles in kaempferol neuroprotective effect In addition, kaempferol regulates various proinflammatory cytokines (TNF-α, IL-6, IL-1β, and IL-18) in addition to the inflammation-related singling pathways including NLRP3, MAPK, NF-κB inflammasome pathways (Cai et al. 2022; Jin et al. 2023).

**Fig. 9:**
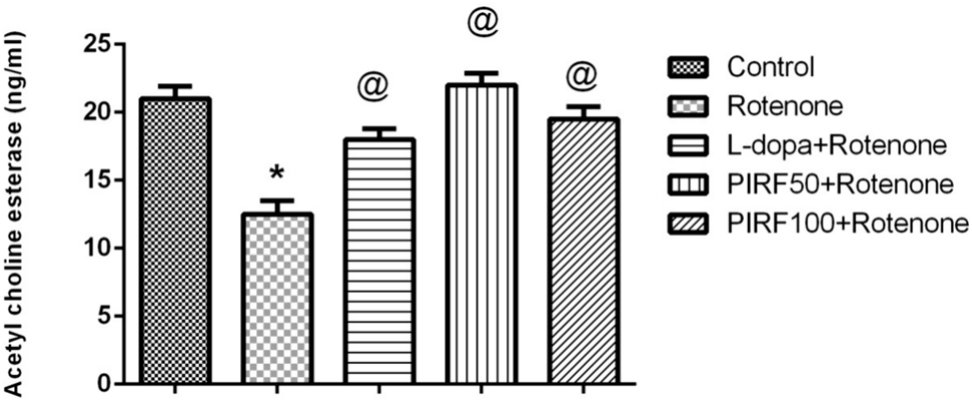
Effect of PIRF treatment in rotenone model of Parkinsonism on acetylcholine esterase level. *, significantly different from control group. Each value represents the mean of 8 values ± standard error of mean (SEM)

Inhibition of acetylcholinesterase (AChE) leads to the accumulation of ACh, which in turn increases muscarinic stimulation and ultimately alleviates memory deficits caused by different neurotoxins (Jin et al. 2023). AChE is one of the targets of most of the clinically used agents for the treatment of dementia (Saad et al. 2024a, b). Cholinesterase inhibitors possess a strategy for the cure of neurodegenerative diseases viz Alzheimer's and Parkinson's (Saad et al. 2024a, b). The enzyme acetylcholine esterase (AChE) is the key enzyme in the hydrolysis of the neurotransmitter acetylcholine and is also the target of most of the clinically used agents for the treatment of PD (Wang et al. 2020). AChE level in brain homogenates of different treated groups was represented in Fig. 9. Rotenone treatment had decreased the AchE level by 40.5%. This depletion was restored to normal levels using both L-dopa and PIRF. Seven iridoids and one flavonoid were isolated from PIRF. Results of studies suggested that iridoids ameliorated anxiety and depression-like behavior in line with what was described by adaptogens and immunomodulators (Wang et al. 2020; Abd-Alla et al. 2021).

The anti-inflammatory benefits of iridoids are linked to their antioxidant properties (Wang et al. 2020; Abd-Alla et al. 2024). These characteristics have the ability to shield tissues and cells from oxidative stress and inflammation-induced apoptosis. The iridoids structure may have an impact on their capacity to treat inflammation (Sweelam et al. 2017; Abd-Alla et al. 2024). Iridoids can protect the cells from harm and modulate the inflammatory response due to their structure. Iridoids have shown a variety of pharmacological effects including anti-inflammatory, antioxidant and neuroprotective in various neurodegenerative disorders (Jaafar et al. 2024). Conducting more research on this class of compounds as leads for future drug discovery toward neurodegenerative disorders including PD is recommended by the present study. Other bioactive components are the flavonoids. Flavonoids with more hydroxyl groups exhibited a greater inhibition on AChE (Jin et al. 2023). By successfully lowering inflammatory markers such IL 6, NO, and TNF-α, the flavonoids such as kaempferol diglycoside demonstrate excellent anti-inflammatory capabilities (Mohamed et al., 2014). Because they can eliminate reactive oxygen species (ROS), these flavonoids have strong anti-oxidant and anti-inflammatory capabilities (Awad et al. 2014; Saad et al. 2024a, b).

**Fig. 10:**
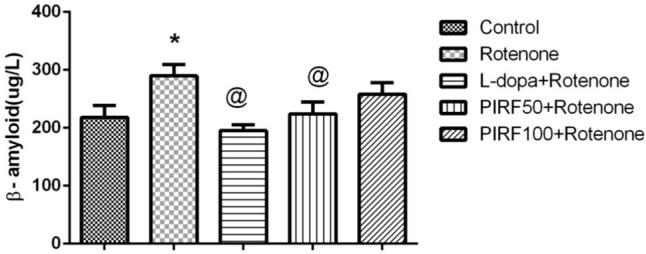
Effect of PIRF treatment in rotenone model of Parkinsonism on β- amyloid content. *, significantly different from control group. Each value represents the mean of 8 values ± standard error of mean (SEM)

The content of β-Amyloid in brain homogenate of different groups was shown in Fig. 10. This content was increased by 33% due to treatment with rotenone. Treatment with L-dopa and the iridoids-rich fraction isolated from *Pentas lanceolata* PIRF leaves restored the β-amyloid content to its normal level as in the control group.

**Fig. 11:**
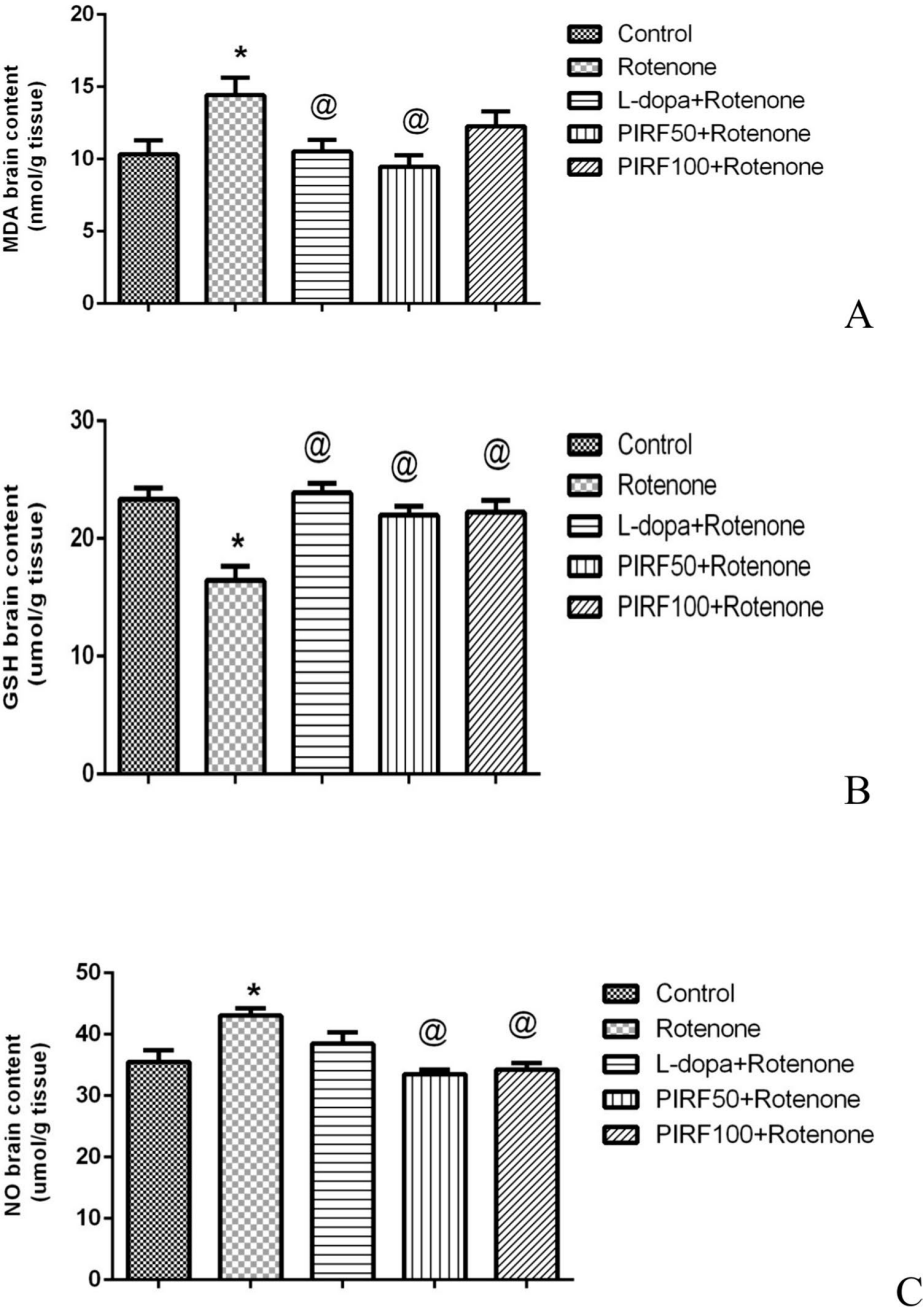
Effect of PIRF treatment in rotenone model of Parkinsonism of oxidative markers. **A** on MDA brain content, **B** on GSH brain content, and **C** on NO content. *, significantly different from control group. @, significantly different from rotenone group. Each value represents the mean of 8 values ± standard error of mean (SEM)

Determination of thiobarbituric acid reactive substances is based on the degradation of lipid peroxides as MDA. MDA content in brain homogenates of different treated groups was represented in Fig. 11A. Rotenone treatment had elevated MDA content by 26.8%. This elevation was restored to normal value using both L-dopa and the iridoids-rich fraction isolated from *Pentas lanceolata* PIRF leaves (50 & 100 mg/kg). The reduced glutathione content in brain homogenates of different treated groups was represented in Fig. 11B. Rotenone treatment depleted GSH content by 29.53%. This decrement in GSH content was restored to normal value using both L-dopa and PIRF.

The content of nitric oxide NO in brain homogenates of different treated groups was represented in Fig. 11C. Rotenone treatment had increased NO content by 21.23%. This elevation reversed to normal value using both L-dopa and the iridoids-rich fraction isolated from *Pentas lanceolata* PIRF leaves (50 & 100 mg/kg).

### Histopathological results

The brains from all experimental were fixed in 10% neutral buffered formalin. In an ascending series of ethanol, the fixed samples were dehydrated. The samples were cleared in zylene and embedded in paraffin wax. Sections 5 µm thickness was prepared using a microtome. The routine hematoxylin and eosin (H and E) stain was used for staining and examination under a light microscope was carried out.

#### Histopathologic study

**Fig. 12:**
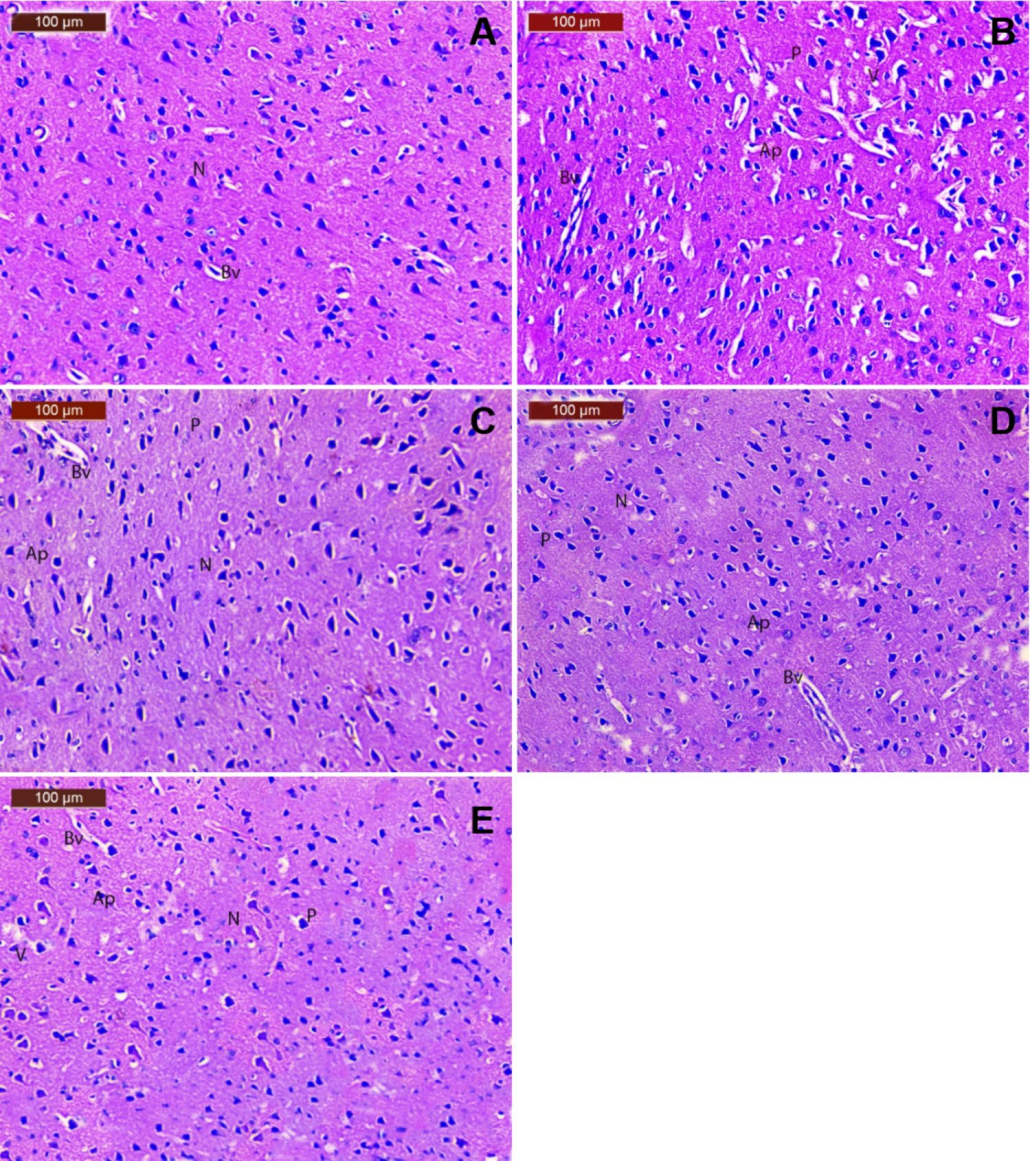
A photomicrograph of brain. **A** control group shows normal histological structure brain tissue with normal neurons (N), **B** rotenone treated group shows disorganization of striatum, degenerated neurocytes with dilated blood vessels (Bv), vacuolation (V) pyknotic nuclei (P), apoptotic cells (Ap), (C): rotenone and reference L-dopa treated group shows moderate improvement with perivascular vacuolation (V), pyknotic nuclei (P), apoptotic cells (Ap) and slight with dilated blood vessels (Bv), (D): rotenone and low dose PIRF treated group shows moderate improvement was seen with slight perivascular vacuolation (V), pyknotic nuclei (P), apoptotic cells (Ap) and slight with dilated blood vessels (arrowhead), (E): rotenone and high dose PIRF treated group shows noticeable improvement was seen in almost neuronal cells of striatum, with few histopathological changes such as minimal pyknotic nuclei (P), apoptotic cells (Ap) and normal dilated blood vessels (Bv)

The brain sections from the control group showed normal architecture of striatum with neurons being arranged in neat rows with abundant cytoplasm, and the nuclei are round (Fig. 12A). Histopathological examination of brain treated with rotenone showed disorganization of striatum, degenerated neurocytes with dilated blood vessels, slight vacuolation, shrunken neurons with pyknotic nuclei and apoptotic cells (Fig. 12B). In the group treated with rotenone and reference drug showed moderate improvement in neuronal cells was seen with perivascular vacuolation, pyknotic, apoptotic cells and slight dilated blood vessels (Fig. 12C). In the group treated with rotenone and low dose of drug, moderate improvement was seen in neuronal cells with slight perivascular vacuolation, pyknotic nuclei, apoptotic cells and slight with dilated blood vessels (Fig. 12D). In the group treated with rotenone and high dose of drug showed noticeable improvement was seen in almost neuronal cells of striatum, with few histopathological changes such as minimal pyknotic nuclei, apoptotic cells and normal dilated blood vessels (Fig. 12E).

## DISCUSSION

PD is one of the most progressive incurable neurodegenerative diseases (Dinda et al. 2019). The disease affects millions of people worldwide. Their treatment is a challenging task of clinical medicine and basic science. PD deteriorates behavioral functions and significantly damages the quality of life (Dinda et al. 2019). PD is mainly associated with oxidative stress accompanied by intraneuronal accumulation of α-synuclein protein aggregates and apoptosis of dopaminergic neurons (Singh et al. 2023; Zhou et al. 2023). The currently used treatments for PD are only symptomatic and do not stop the sequel of the disease. The strategy for developing novel neuroprotective drugs aims to restoration of normal brain function in PD patients and improvement of neuronal cell loss (Dinda et al. 2019). Rotenone is a classical mitochondrial complex inhibitor with high affinity to induce parkinsonian like symptoms (Grover et al. 2023). Long-term administration of such neurotoxic compound increases oxidative stress by releasing excess ROS leading to mitochondrial dysfunction in the striatum and cortex, and abnormality in the nigrostriatal dopaminergic neurons (Burke and O'Malley, 2013).

Recently, natural herbs have been the focus of many studies because they possess potential bioactive components for preventing or treating diseases of neurodegenerative nature and have beneficial effects on the aging brain (El-Baz et al., 2018; El-Shamarka et al. 2020, 2023; Saad et al. 2024a, b).

Iridoids are secondary plant metabolites that are multitarget compounds active against various diseases. Biologically active iridoid derivatives have been found in a variety of plant families, including Verbenaceae, Plantaginaceae, Scrophulariaceae, and Rubiaceae (Sharma et al., 2019; Grover et al. 2023; Abd-Alla et al. 2024). More than 3000 kinds of iridoids have been isolated and identified from plants according to incomplete statistics. They are structurally classified into iridoid glycosides and non-glycosidic iridoids according to the presence or absence of intramolecular glycosidic bonds; additionally, iridoid glycosides can be further subdivided into carbocyclic iridoids and secoiridoids (Sweelam et al. 2017; Wang et al. 2020). More than 30 kinds are secoiridoids, 60 kinds are non-glycoside iridoids, and most of which are glycosides (Wang et al. 2020; Zhou et al. 2023). Many naturally occurring monoterpenoids; iridoids and their glycosides have variety of pharmacological functions such as antioxidant, anti-inflammatory, and neuroprotective and neurotrophic effects (Zhou et al. 2023; Abd-Alla et al. 2024). Plant iridoids such as geniposide, loganin, 10-*O*-*trans*-*p*-coumaroyl derivative, harpagoside, and catalpol have been reported with the property of slowing down the process of neurodegeneration and improving the neuroprotective effect in PD (Tseng et al. 2019; Wang et al. 2020).

Regulations in cellular reduction/oxidation (redox) processes are being increasingly implicated in PD, and antioxidant agents are aimed at a promising pathway to treatment (Wang et al. 2020; Zhou et al. 2023). Researches support that variant secondary metabolites with antioxidant properties are promising agents ameliorate the oxidative damage-induced neurotoxicity by suppressing oxidative stress (Awad et al. 2014; Abd-Alla et al. 2022; Aziz et al. 2022).The effect of iridoid glycoside catalpol in an animal model of PD has been studied and the results finding reported that this compound can effectively improve rotenone-induced oxidative stress (Zhang et al. 2021). Secondary metabolites of iridoids such as catalpol and loganin were found to dose-dependently protect midbrain neurons from neurotoxin-induced oxidative stress, especially dopaminergic neurons (Tseng et al. 2019; Wang et al. 2020).

Recent studies (in vivo and in vitro) on the pharmacological mechanism of variant iridoids have shown that they may exert anti-PD effects through multiple mechanisms (Wang et al. 2020; Zhang et al. 2021; Abd-Alla et al. 2024). For example, through the mechanism of blocking microRNA-21/lysosome-associated membrane protein 2A interaction, the iridoid geniposide has been reported to reduce α-synuclein in PD model (Zhou et al. 2023; Grover et al. 2023). Investigation of the phytochemical study of PIRF resulted in the isolation of a kaempferol derivative (compound **8**). Kaempferol has reported to increase the expression of tyrosine hydroxylase TH in rotenone-induced PD flies, bound to human α-synuclein, and reduce oxidative stress markers, suggesting that kaempferol may inhibit the aggregation of α-synuclein (Jin et al. 2023).

Neuronal survival is managed by interrelated network of signaling sequence. An imbalance of such cascade may have dramatic consequences on neuronal growth and differentiation (Akbari et al. 2019). These processes are predominantly upregulated by one of the most significant factors such as Brain Derived Neurotrophic Factor (BDNF), which is vital for dopaminergic neuronal persistence, plasticity, and differentiation (Miranda et al. 2019).

Motor impairment, the result of dopaminergic cell injury is one of the neuropathological hallmarks of PD (Dijkstra et al. 2014). In agreement with other studies (Singh et al. 2023) it was monitored that mice intoxicated with rotenone showed behavioral changes represented by motor dysfunction including prolonged wooden-walking time and the time taken to climb up the stairs as well as impaired grip strength by reducing the wire-hanging time. While administration of L-dopa or iridoids-rich fraction of *P. lanceolata*, results in the reduction of latency time in the wooden-walking test as well as a less time is consumed by the mice to reach up the stairs. The grip strength was also improved by treatment with either L-dopa or PIRF 50 and 100 mg/kg by increase in wire-hanging time.

Our present study recorded for first time the isolation of the flavonol diglycoside; compound **8** (kaempferol-3-*O*-robinobioside); a rare class of compounds present in the genus of *Pentas*. Kaempferol significantly alleviated cognitive impairment and behavioral abnormalities, in a valium-induced memory impairment ICR model of mouse. In addition to anti-cholinesterase effects, kaempferol has anti-oxidant and anti-inflammatory activity. Also, kaempferol was speculated to act on the esterification subunit of AChE or ionic site and inhibits AChE activity for this reason, although its mechanism for improving of memory impairment was unclear (Jin et al. 2023). Kaempferol diglycosides have been reported to restored TH activity and reduce ROS levels, suggesting that these derivatives are useful lead compounds for PD therapy (Jin et al. 2023). Also, in PD mice, it was reported that kaempferol improved dopamine metabolite levels, increased the number of dopaminergic cells, and ameliorated behavioral deficits (Jin et al. 2023).

In the study, seven iridoids (**1**–**7**) were isolated from* P. lanceolata* iridoid-rich fraction PIRF. Our findings suggest that PIRF treatment significantly enhanced grip strength and motor discoordination induced by rotenone intoxication. The protective effects of iridoid glycosides against Parkinson's disease mimetic toxin 1-methyl-4-phenylpyridinium (MPP^+^) was reported (Tseng et al. 2019). An enhancement of the neurotrophic signals expression through up regulating the expressions of glucagon-like peptide 1 receptor (GLP-1R), p-Akt and tyrosine hydroxylase were suggested. To reduce MPP^+^-induced neuron damage, it can reduce the production of MPP^+^-induced ROS and down-regulate membrane-rhoA/ROCK2/p-LIMK/p-cofilin and up-regulate GAP43 (Tseng et al. 2019). The iridoids could enhance neurotrophic signals, activate IGF-1R/GLP-1R, and other mechanisms such as inhibit RhoA/ROCK pathway of neuron damage (induced by 1-methyl-4-phenylpyridinium), to achieve neuroprotective action (Tseng et al. 2019).

A secoiridoid glycoside; swertiamarin (from *Enicostemma littorale* Blume) is a neuroprotective agent and possesses a strong anti-inflammatory effect. The rotenone-induced α-syn overexpression in the substantia nigra (SN) and striatum were alleviated by this metabolite (Sharma et al. 2022). Suppression of microglial activation and restoration of the neuroprotective effect were exhibited after administration of this secoiridoid glycoside in a rotenone mouse model (Abdel-Salam et al. 2014). In the nigrostriatal pathway, the compound mitigated the loss of dopaminergic neurons and ameliorated the motor impairment induced by rotenone (Sharma et al. 2022). The extract from the Chinese plant of *Scrophularia ningpoensis* with iridoid glycosides has been used for the treatment of Parkinsonism. The fraction of a plant belonging to Rubiaceae family has neuroprotective properties. In rats’ brain homogenate, the properties were proposed to be associated with their inhibitory effect on Fe^2+^-induced lipid peroxidation and modulation of activities of Na^+^/K^+^-ATPase, monoamine oxidase, butyrylcholinesterase and acetylcholinesterase (Wang et al. 2020). The fraction contains lamalbide 6,7,8-triacetate and its aglycone lamiridosin 6,7,8-triacetate, in a dichloromethane fraction of the plant *Heinsia* *crinita* 's stem. The extract of *Valeriana jatamansi* rich in iridoids demonstrated that it possesses an encouraging neuroprotective effect (Wang et al. 2020). These suggested that the extract with iridoid compounds may be employed to treat CNS disorders, namely anxiety disorder, Alzheimer’s disease, cerebral infarction, and Parkinson’s disease (Wang et al. 2020; Abd-Alla et al. 2024).

Noteworthy, rotenone administration caused a decrease in dopamine brain content and acetylcholinesterase activity as well. Rotenone displayed severe exhaustion in the dopaminergic striatal pathway (Betarbet et al., 2000). A deficiency in presynaptic energy in dopaminergic neurons could be attributed to such depletion detected in the rotenone treated group (Alam and Schmidt 2002). While such depleted effect is improved by L-dopa and PIRF and restored to normal value.

Several findings proved that neuroinflammation is involved in the etiology and pathogenesis of PD (Williams et al. 2018; Hirsch and Standaert 2021). In the brain, NF-κB stimulation enhances the inflammatory responses and the release of pro-inflammatory molecules as TNF-α and IL-1B directly or indirectly in the brains of PD persons compared to controls (Dolatshahi et al. 2021; Saad et al. 2024a, b). It was firmly expected that such proinflammatory cytokines have a significant role in inflammation and neurodegeneration (Mohammad et al. 2023). Moreover, microglial activation induced by rotenone has been related to increased release of TNF-α in the cerebellum and striatum (Farombi et al. 2019). The neuroprotective effect of catalpol has been promoted to an upsurge in recent years, mainly for the prophylaxis and treatment of neurodegenerative diseases including PD (Zhou et al. 2023). This iridoid glycoside reduces inflammatory cytokines in the senescent mice induced by D-galactose and improves cholinergic function (Wang et al. 2020; Zhou et al. 2023; Abd-Alla et al. 2024). Abdel-Sattar et al., 2021 showed that the levels of some proinflammatory cytokines as IL-1B and TNF- ∝ were heightened in rotenone model which is in harmony with our results. Moreover, the current study confirmed this finding that rotenone (orally administrated)-enhanced the release of IL-1B and TNF- ∝ in the cerebellum and striatum. This is possibly due to activation of microglia caused by rotenone revealing its neuroinflammation. However, rotenone-induced neuroinflammation was attenuated by the iridoids-rich fraction isolated from *P. lanceolata* (PIRF) leaves and L-dopa confirming their anti-inflammatory and neuroprotective effects. As previously mentioned, numerous evidence reveal that PD is associated with aggravated inflammatory cascade and damage (Badawi et al. 2020; Tansey et al. 2022). This is in accordance with observed enhancement in MCP-1 in the rotenone-treated group, an effect that was ameliorated with PIRF and L-dopa, in the current investigation.

Several signaling pathways have been implicated in the development of PD, with α-synuclein insertion adopting a β-sheet-rich amyloid-like form being the key to the succession of the disease. Our results reported that the β-amyloid content has been increased in brains treated with rotenone. On the other hand, treatment with L-dopa and PIRF reversed such increment. These results are in accordance with others (Caviness 2014).

Stressful environment results in free radicals' production which interacts with oxygen molecules on membrane lipids to produce peroxy radicals, which are accountable for lipid peroxidation represented by MDA (Rizk et al. 2018; Aziz et al. 2022). Our research reveals that rotenone-treated mice showed elevated MDA levels, as reported by others (Mohammad et al. 2023; Aleksandrova et al. 2023) whereas such enhancement is restored to normal using L-dopa and the iridoids-rich fraction isolated from *Pentas lanceolata* (PIRF) leaves as results in decrease in MDA level.

It was reported that the progression of PD results from Lewy bodies (LB) that are produced by the insertion of α-synuclein oligomers (Haque et al. 2022). This exerts a destruction of dopaminergic neurons in the substantia nigra (SN) (Ingelsson 2016). An up-regulation of inducible nitric oxide synthase (iNOS) was due to SN mitochondrial presence of α-synuclein. Also, mitochondrial dysfunction was produced as a result of the production of ROS and mitochondrial membrane potential deficits (Haque et al. 2022). Accordingly, Zhang et al. 2018 explored that rotenone induced neuroinflammation via significant rise in levels of NO. This finding supports our results which reveals that nitric oxide (NO) content in the brain is significantly increased after rotenone treatment while this elevation has been reversed to normal value with L-dopa and the iridoids-rich fraction isolated from *Pentas lanceolata* (PIRF) leaves.

In the nerve cells, an oxidative damage could be induced by the elevated levels of ROS which can lead to radical-induced cell death and neuronal injury (Abd-Alla et al. 2022; Theofanous and Kourti 2022). Reduced activity of some major antioxidants was observed in previous studies which reported that rotenone administration caused an increase in acetylcholine esterase and lipid peroxidation activity. A decrease in the level of GSH, SOD, and catalase as antioxidant enzymes in the prefrontal cortex, striatum and cerebellum are indicative of cholinergic dysfunction and oxidative stress (Birla et al., 2021, Ishola et al. 2023). These findings are in parallel with ours which evidenced that rotenone treatment had depleted GSH content. This decrement in GSH content was reversed using both L-dopa and PIRF.

Neuropathological changes have been found in recent research related to PD (Sharma et al. 2022; Zhou et al. 2023). The present study explored that histopathological examination of brain treated with rotenone showed disorganization of striatum, degenerated neurocytes with dilated blood and shrunken apoptotic cells. While animals treated with rotenone and high dose of PIRF showed noticeable improvement in the brain neuronal striatum of affected animals. Ultimately, fractions or extracts of plants rich in iridoids may be candidate drugs for the prophylaxis or treatment of neurodegenerative diseases such as PD (Zhou et al. 2023; Abd-Alla et al. 2024). Fraction containing naturally occurring monoterpenoids; iridoids have potential activities against PD in animal and cellular models. The isolation and structural elucidation of these secondary plant metabolites are still a huge and fast growing approach by authors for future study in this field.

## Conclusion

In treatment and/or prevention of PD, the potential neuroprotective activity of some iridoids-rich fraction of plants is highlighting their key molecular targets in neuroprotection of this disease, with an aim for their application as low-cost eco-friendly drugs in future.

## Supplementary Information

Below is the link to the electronic supplementary material.

Supplementary file1 (DOCX 3525 kb)

## Data Availability

The datasets generated during and/or analysed during the current study are available from the corresponding author on reasonable request.
