# Supplementary material for: Chemical composition and studying the possible neuroprotective effect of iridoids-rich fraction from Pentas lanceolata leaves using rotenone model of Parkinson’s disease in mice
Source: Inflammopharmacology. 2024 Jul 4;32(6):3953–71. doi: 10.1007/s10787-024-01509-9 (PMC11550285; doi:10.1007/s10787-024-01509-9)
Supplement: Supplementary file 1 — Supplementary file1 (DOCX 3525 kb) [file 10787_2024_1509_MOESM1_ESM.docx]

**Table of Contents**

| Supporting Figure S1. ^1^H NMR spectrum of compound 1 in CD_3_OD | **2** |
| --- | --- |
| Supporting Figure S2. ^13^C-NMR spectrum of compound 1 in CD_3_OD | **3** |
| Supporting Figure S3. ^1^H NMR spectrum of compound 2 in CD_3_OD | **4** |
| Supporting Figure S4. ^13^C-NMR spectrum of compound 2 in CD_3_OD | **5** |
| Supporting Figure S5. ^1^H NMR spectrum of compound 3 in CD_3_OD | **6** |
| Supporting Figure S6. ^13^C-NMR spectrum of compound 3 in CD_3_OD | **7** |
| Supporting Figure S7. ^1^H NMR spectrum of compound 4 in CD_3_OD | **8** |
| Supporting Figure S8. ^13^C-NMR spectrum of compound 4 in CD_3_OD | **9** |
| Supporting Figure S9. ^1^H NMR spectrum of compound 5 in CD_3_OD | **10** |
| Supporting Figure S10. ^13^C-NMR spectrum of compound 5 in CD_3_OD | **11** |
| Supporting Figure S11. ^1^H NMR spectrum of compound 6 in CD_3_OD | **12** |
| Supporting Figure S12. ^13^C-NMR spectrum of compound 6 in CD_3_OD | **13** |
| Supporting Figure S13. ^1^H NMR spectrum of compound 7 in CD_3_OD | **14** |
| Supporting Figure S14. ^13^C-NMR spectrum of compound 7 in CD_3_OD | **15** |
| Supporting Figure S15. ^1^H NMR spectrum of compound 8 in CD_3_OD | **16** |
| Supporting Figure S16. ^13^C-NMR spectrum of compound 8 in CD_3_OD | **17** |


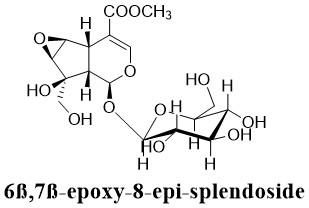


**Figure S1.** ^1^H NMR spectrum (600 MHz, CD_3_OD) of compound **1**


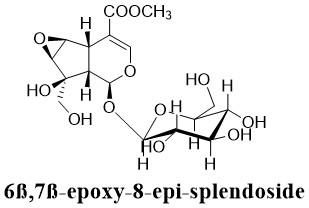


**Figure S2.** ^13^C NMR spectrum (150 MHz, CD_3_OD) of compound **1**


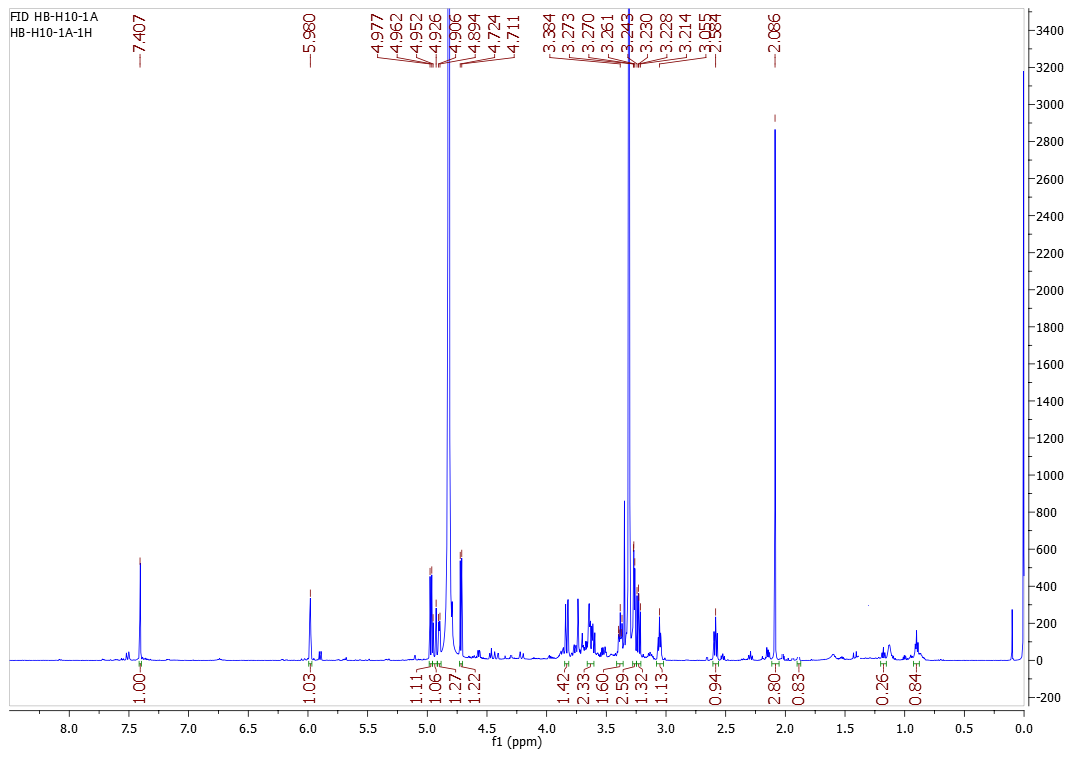

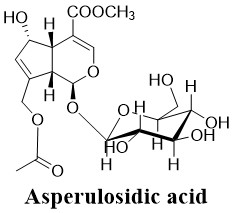


**Figure S3.** ^1^H NMR spectrum (600 MHz, CD_3_OD) of compound **2**


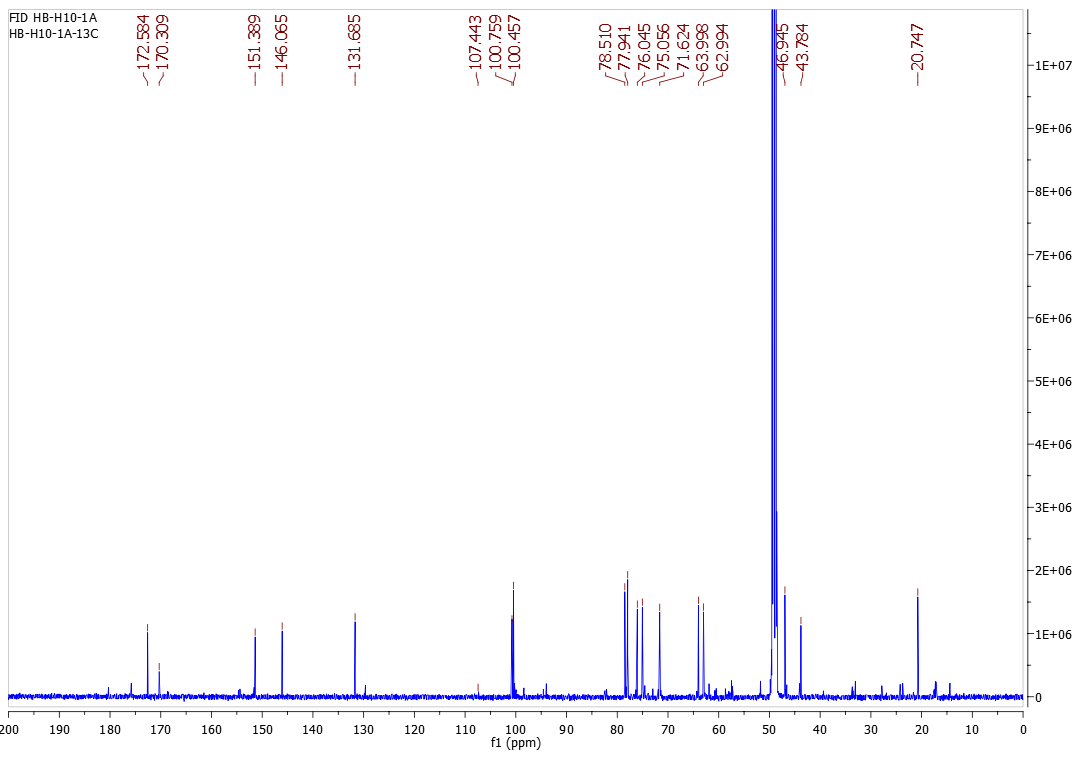

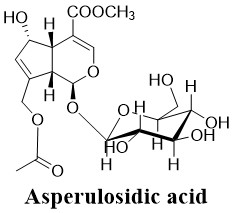


**Figure S4.** ^13^C NMR spectrum (150 MHz, CD_3_OD) of compound **2**


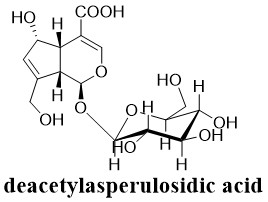


**Figure S5.** ^1^H NMR spectrum (600 MHz, CD_3_OD) of compound **3**


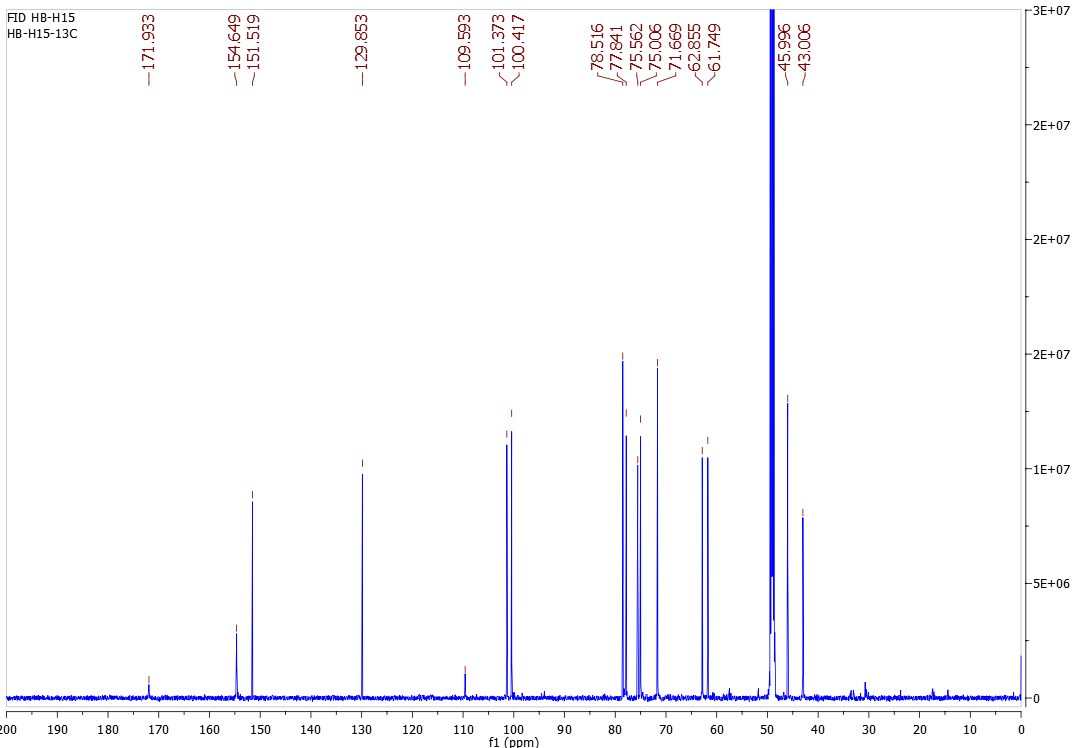

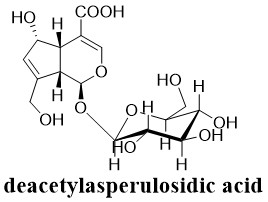


**Figure S6.** ^13^C NMR spectrum (150 MHz, CD_3_OD) of compound **3**


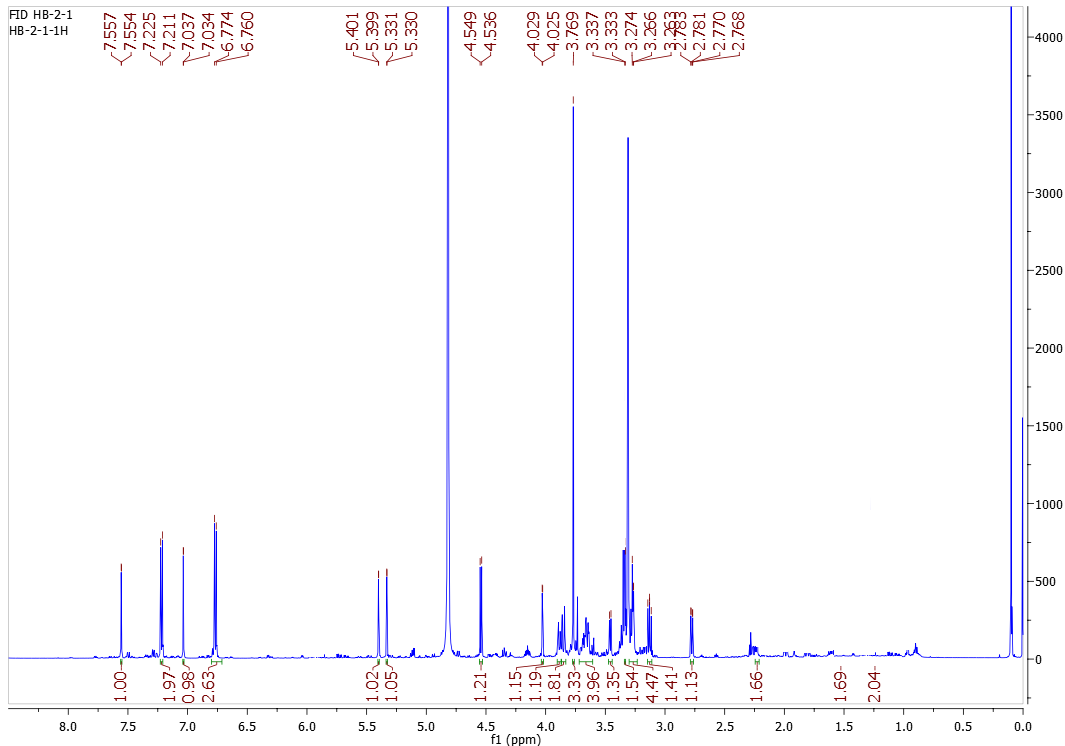

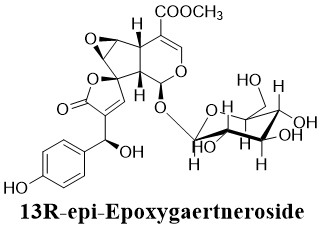


**Figure S7.** ^1^H NMR spectrum (600 MHz, CD_3_OD) of compound **4**


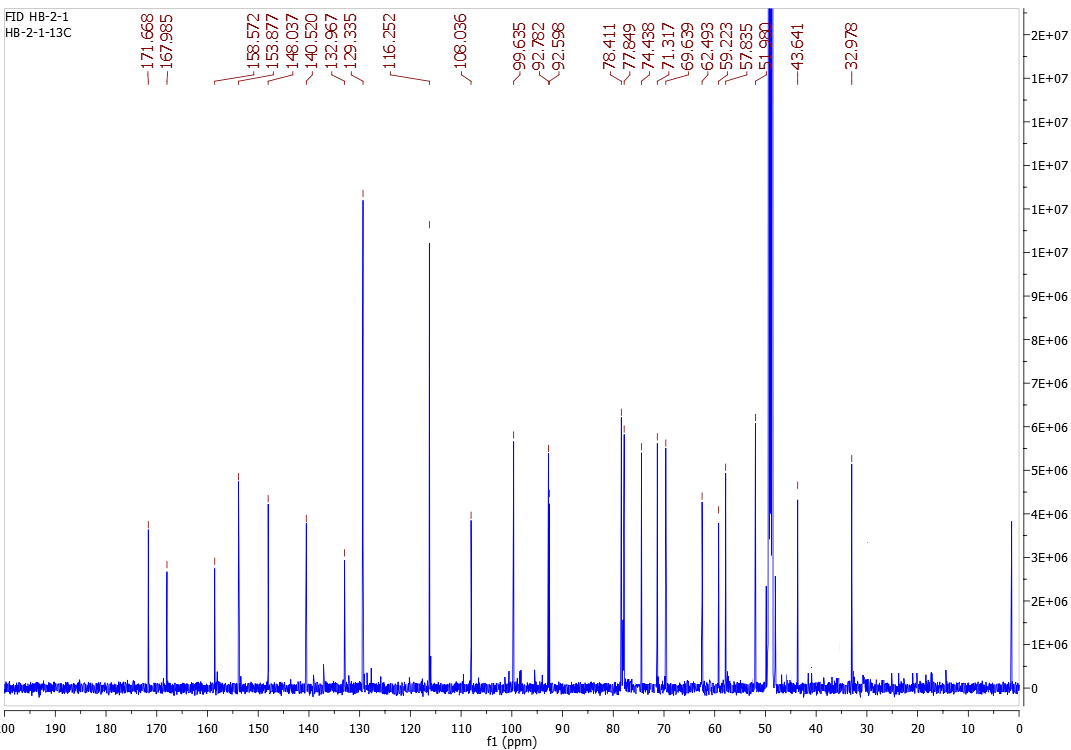

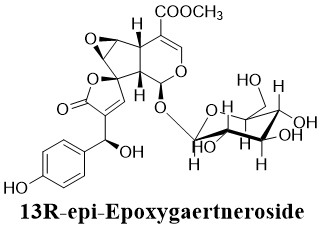


**Figure S8.** ^13^C NMR spectrum (150 MHz, CD_3_OD) of compound **4**


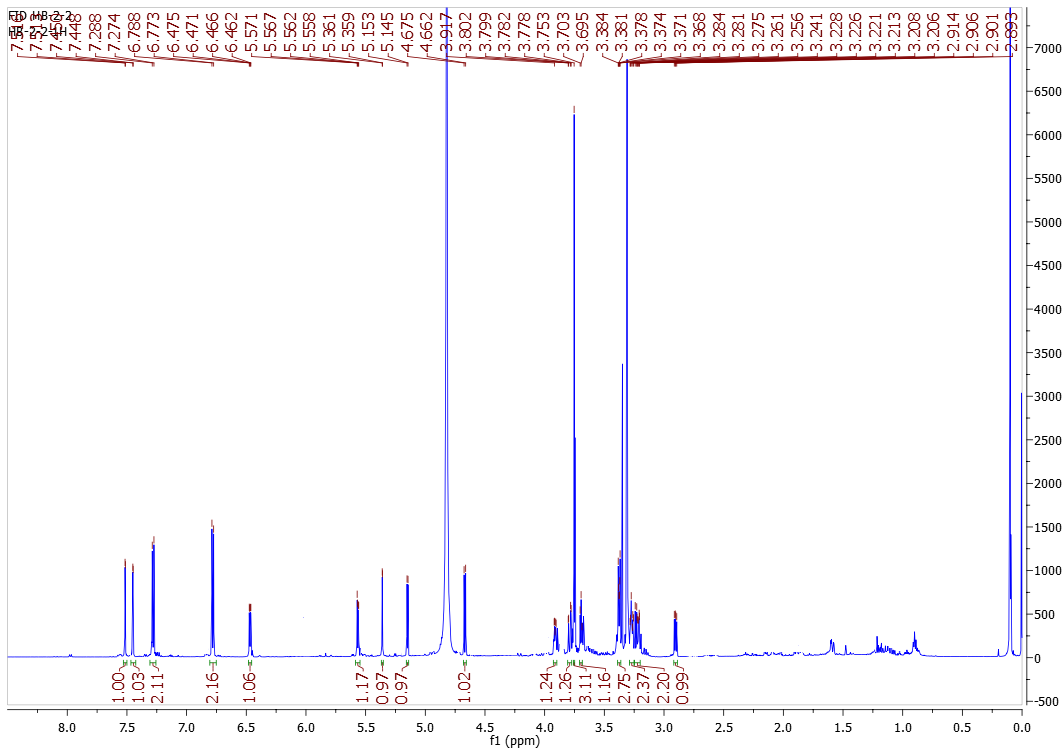

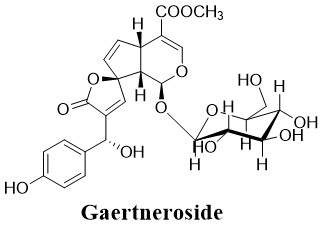


**Figure S9.** ^1^H NMR spectrum (600 MHz, CD_3_OD) of compound **5**


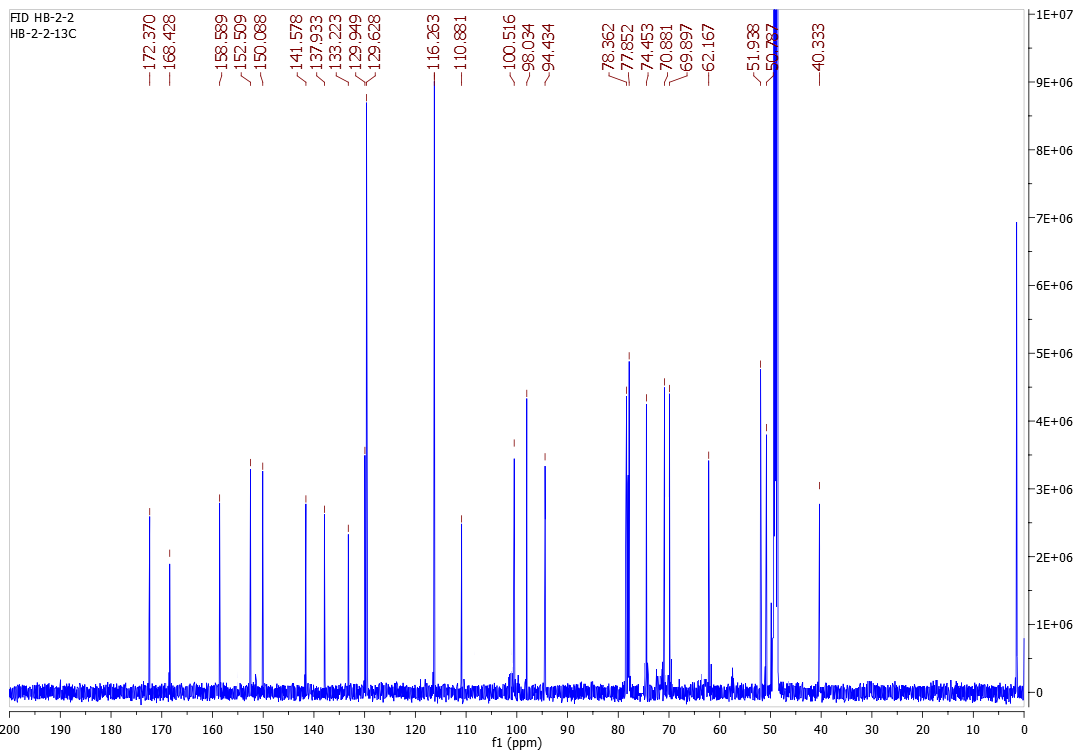

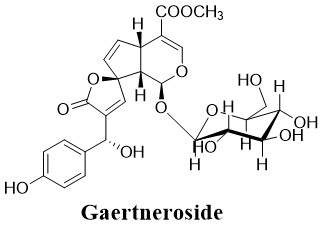


**Figure S10.** ^13^C NMR spectrum (150 MHz, CD_3_OD) of compound **5**


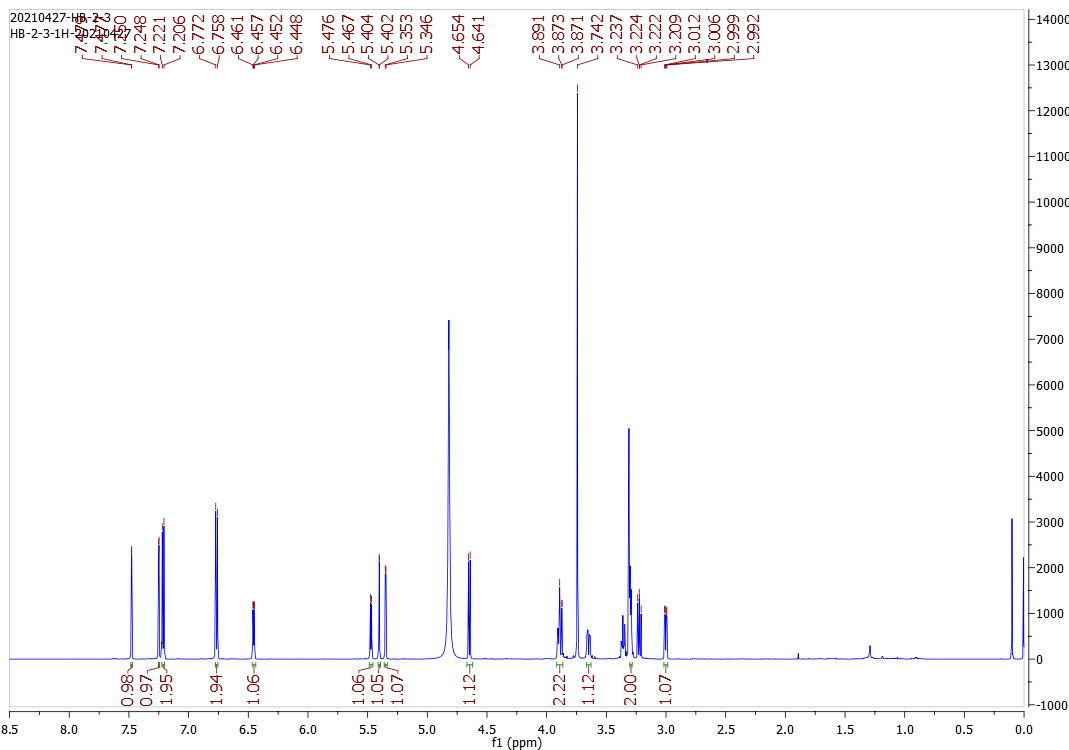

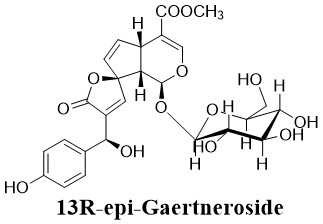


**Figure S11.** ^1^H NMR spectrum (600 MHz, CD_3_OD) of compound **6**


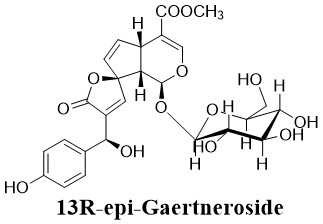


**Figure S12.** ^13^C NMR spectrum (150 MHz, CD_3_OD) of compound **6**


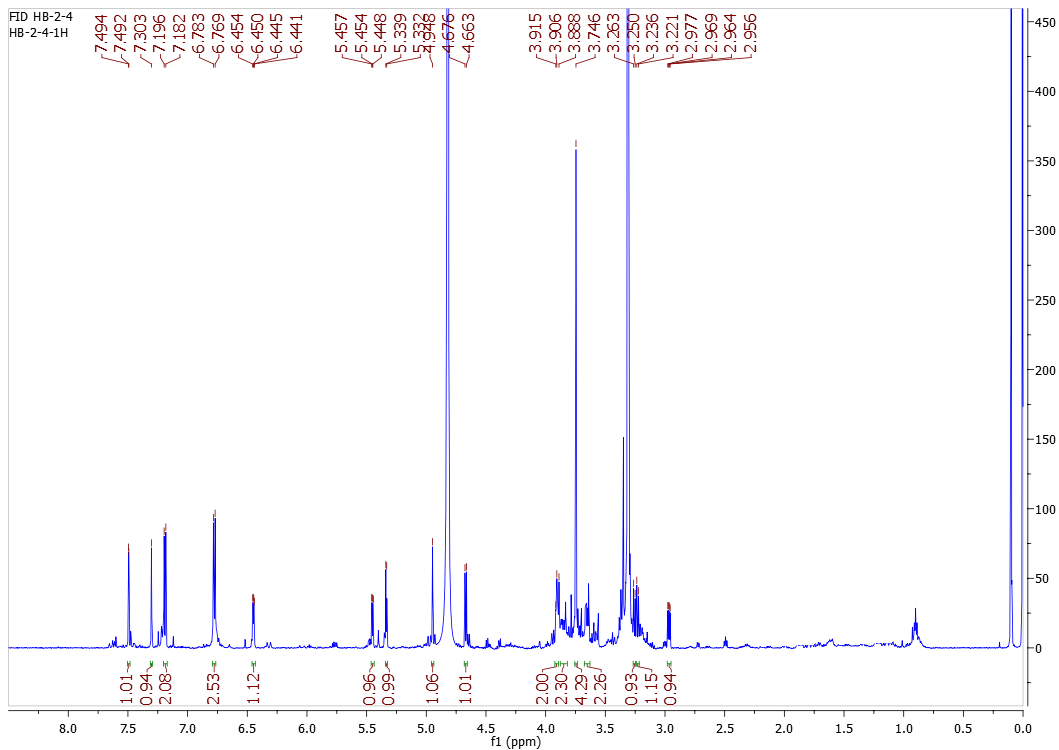

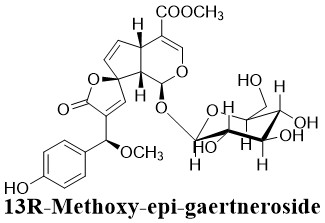


**Figure S13.** ^1^H NMR spectrum (600 MHz, CD_3_OD) of compound **7**


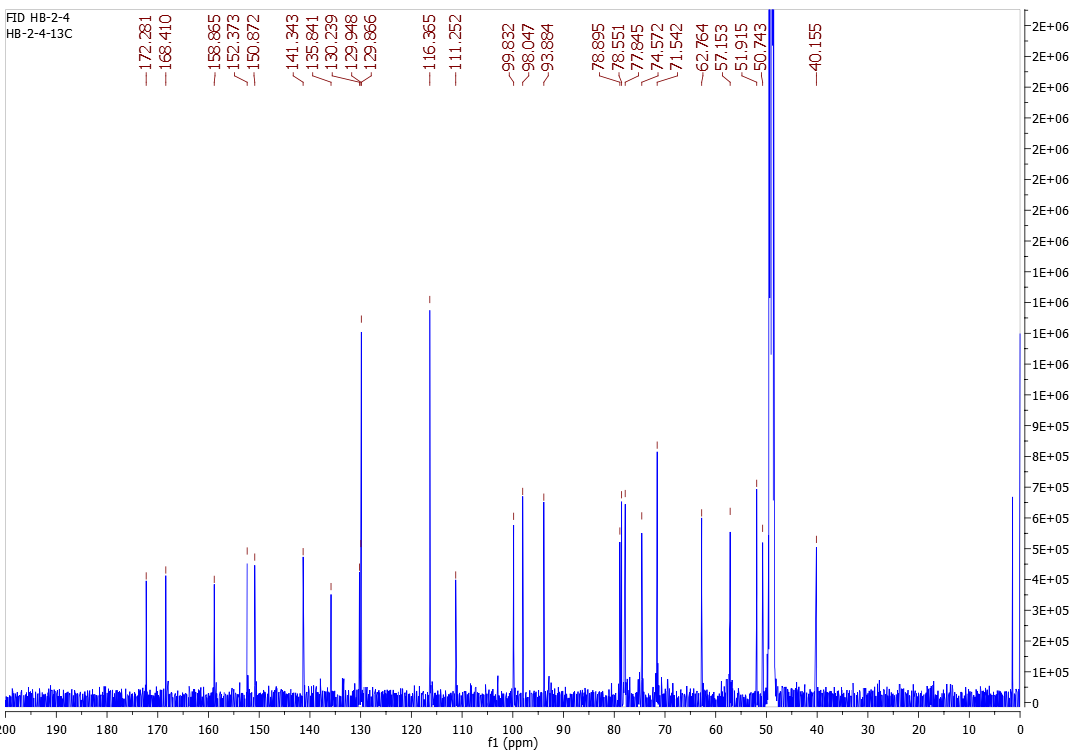

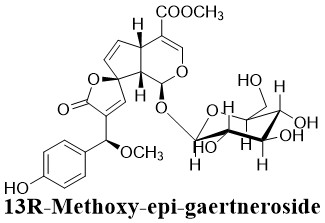


**Figure S14.** ^13^C NMR spectrum (150 MHz, CD_3_OD) of compound **7**


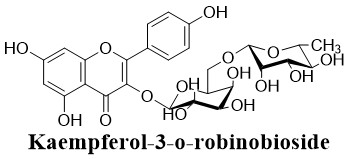


**Figure S15.** ^1^H NMR spectrum (600 MHz, CD_3_OD) of compound **8**


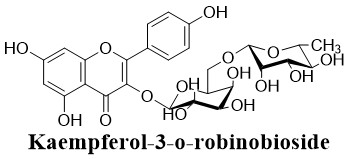


**Figure S16.** ^13^C NMR spectrum (150 MHz, CD_3_OD) of compound **8**
